# Sharing meals is associated with greater wellbeing

**DOI:** 10.1038/s41598-026-46771-9

**Published:** 2026-04-22

**Authors:** Jan-Emmanuel De Neve, Andrew Dugan, Micah Kaats, Alberto Prati

**Affiliations:** 1 University of Oxford, Oxford, UK; 2 Gallup, Washington, DC, USA; 3 Harvard Kennedy School, Cambridge, USA; 4 University College London, London, UK

**Keywords:** Wellbeing, Sharing, Subjective wellbeing, Food, Social connections, Gallup World Poll, Human behaviour, Quality of life

## Abstract

Although social connections are important drivers of wellbeing, and sharing meals is a nearly universal practice, the relationship between sharing meals and wellbeing remains underexplored. In Study 1, using novel data from Gallup surveys in 142 countries and territories (2022–2023), we use the frequency of meal sharing as a global measure of social connections, and investigate its relationship with subjective wellbeing. We find a significant, positive relationship in almost all world regions. The number of shared meals explains as much variation in wellbeing as some key socioeconomic indicators, such as income or unemployment. In Study 2, we use data from the American Time Use Survey (2003–2023) to document changes in meal sharing in the United States over time. We find clear evidence that, with every passing year, Americans are dining alone more and more often, particularly the younger generations. Using Day Reconstruction Method data, we show that Americans who eat at least one meal with others report higher levels of happiness and lower levels of stress, pain and sadness on that day. Finally, we point to a number of promising avenues for future research and discuss implications for policy.

**Supplementary Information:**

The online version contains supplementary material available at 10.1038/s41598-026-46771-9.

## Introduction

Social connections are critically important for human health, happiness, and prosperity. More socially connected people tend to be happier, less stressed, more satisfied with their lives, less prone to depression, more engaged in their communities, and less likely to suffer from disease or disability^1–5^. In their professional lives, people with more social connections are more creative, cooperative, trusting, and likely to be promoted^6–10^. They are less likely to commit crimes, earn higher levels of income, and live longer lives^2,3,6,11–14^.

At the same time, social isolation and loneliness are strongly associated with negative life outcomes. The absence of social ties has been linked to higher rates of disease, shorter life expectancies, lower levels of subjective wellbeing, higher rates of criminality, and greater support for authoritarianism^6,11–13,15–20^. One widely cited meta-analysis estimated that the negative health consequences of loneliness and isolation were roughly equivalent to smoking 15 cigarettes a day^15,21^.

Social connections are not only important for individual health and happiness, but also for the wellbeing of society writ large. People who are more connected to each other are more trusting of others and have more confidence in institutions^22,23^. They are more likely to donate to charity, be more politically engaged, and report higher levels of pride in their communities^24–31^. They tend to be more considerate and compassionate, not only towards friends and family, but also towards strangers. They are more likely to volunteer time to help those in need and share resources with others^26,28,29,31–33^.

In this paper, we use the frequency of sharing meals as a proxy for social connectedness. Although the topic of sharing meals has remained relatively understudied in the academic literature, the link between food and social relationships is far from new. In French, *copain* (friend) and in Italian *compagno* (mate) come from the Latin *cum* + *pānis*, literally “with-bread”. The Chinese term for companion/partner, 伙伴, stems from a similar term (火伴) which literally translates to “fire mate”, a reference to sharing meals over a campfire.

The number of meals shared with others has a number of advantages compared to existing proxies for social connections. Unlike measures that capture general feelings about social connectedness (such as asking survey respondents about how close they feel to their friends or family or how often they feel isolated or left out), these questions about sharing meals focus on concrete experiences in the past week, making them less prone to certain types of survey bias. Additionally, by measuring a tangible behaviour, these questions have the potential to provide important insights for researchers and policymakers seeking not only to understand the role of social connections but also to identify actionable ways to improve this facet of people’s lives. 

For example, survey respondents may be asked to report how close they feel to friends and family, how many close relationships they have in general, or how often they feel isolated or left out. While undeniably valuable, these sorts of subjective questions present a series of challenges for researchers studying the relationship between subjective measures of wellbeing and subjective indicators of social connection. For one, statistical correlations between all subjective measures tend to be artificially high to begin with. This can lead to potentially misleading interpretations about just how related two subjectively reported variables really are. Moreover, it is also often difficult to assess whether one person’s self-report of a close social connection is directly comparable to someone else’s. These problems are exacerbated when trying to make comparisons across countries and cultures, or over long periods of time.

A metric based on the number of shared meals is well-positioned to address these issues. Sharing meals is a cross-cultural social ritual, practiced every day by billions of people. It is a universal practice. This is particularly useful when studying social connections and wellbeing on a global scale as it allows for relatively reliable international and intercultural comparisons. The number of meals shared with others is also much more objectively comparable over time than related measures of social engagement. While interpretations of closeness or belonging may evolve and change over time, the number of meals shared with others is not expected to. In this way, our approach is operationally similar to other well-established questions to measure related aspects of human capital. For example, the question "How many books were there in your home when you were 16?" is routinely used by international surveys to measure parental cultural capital, and, the World Inequality Study, the OECD’s Programme for the International Assessment of Adult Competencies (PIAAC), and the Life in Transition Survey (LITS) run by the European Bank for Reconstruction and Development all employ versions of this question.

Recently, an emerging body of empirical evidence has begun to point to potential links between sharing meals and a range of social benefits. One review of literature found that adolescents who ate more meals with family members had better diet and nutritional habits, lower levels of obesity, fewer eating disorders, and greater academic achievement^34^. Another experiment found strong links between meal sharing and positive affect, although these effects were diminished with increased smartphone use during meal times^35^. Yet another study of roughly 9,000 older adults in China found that sharing meals with others was associated with lower rates of depressive symptoms^36^.

In Study 1, we extend this body of work by looking at the relationship between sharing meals and wellbeing using novel data collected on a global scale. We present evidence from the first ever global dataset on social eating, collected as part of a special module on the Gallup World Poll in 2022 and 2023, which was sponsored by the Japanese corporation Ajinomoto. Nationally representative surveys conducted in over 140 countries asked more than 150,000 people the following two questions: “Thinking about the last 7 days … (i) On how many days did you eat lunch with someone you know? (ii) On how many days did you eat dinner with someone you know?” In addition to this new dataset, in Study 2, we also present new evidence from the American Time Use Survey (ATUS) in the United States on the association between sharing meals, social connections, and wellbeing over time, from 2003 up to 2023.

The aim of this paper is to assess the relationship between sharing meals and subjective wellbeing. Given the strong link between wellbeing and social connections, sharing meals with others may be an important indicator of positive wellbeing. This indeed turns out to be the case. We present the largest and most robust evidence to date showing that sharing meals with others is strongly predictive of greater life evaluations, increased positive affect, and decreased negative affect. We also find that dining alone is at least as (if not even more) strongly associated with low levels of wellbeing.

Of course, studying the correlation between sharing meals and wellbeing raises an important issue of causality. Does sharing meals make people happier? Or are people who are happy to begin with more likely to share meals? Or, perhaps most likely, is the relationship bi-directional? These are important questions with significant implications for research and policy. We do not conclusively resolve them here. While we discuss preliminary evidence and efforts to get at the underlying causal dynamics of meal sharing and subjective wellbeing, arriving at a full answer to this question is a task that remains open to future research.

## Results

### Study 1—meal sharing and wellbeing around the world

**Fig. 1:**
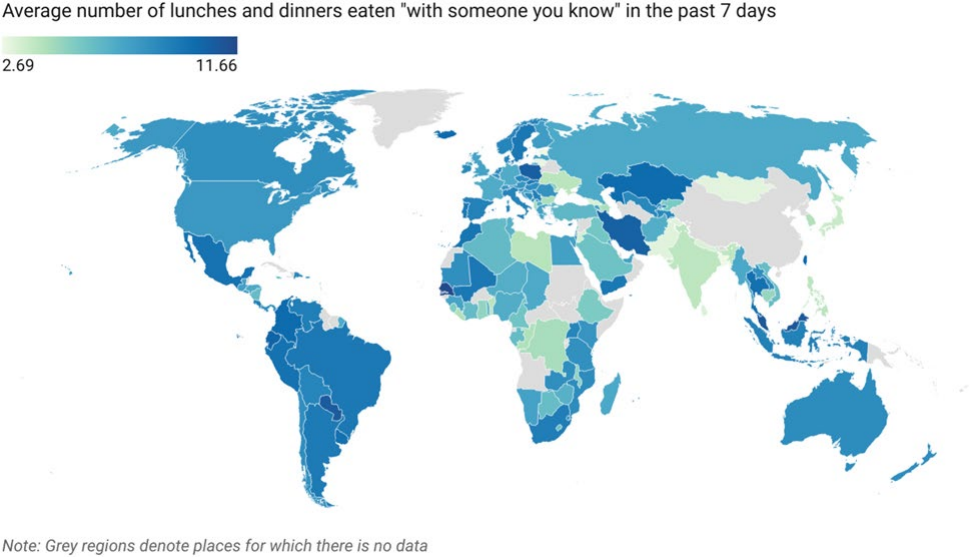
Meal sharing by country. Gallup World Poll, 2022–2023.

In 2022, the Gallup World Poll asked representative samples in 142 countries and territories how often they ate lunch or dinner with family, friends, or anyone else they knew. The same information was also collected in a smaller set of countries in 2023. In Fig. 1, we present rates of meal sharing for all countries. Detailed statistics are reported in the Appendix, Table A1-A2. Overall, we find stark differences in the frequency of dining with others around the world. Latin America emerges as the global leader in meal sharing. On average, Latin Americans share approximately 9 meals with other people per week. At the bottom of the list, the relatively low levels of meal sharing in South and East Asian countries is particularly notable. We consider lunches and dinners together, but the documented patterns are largely consistent when analysing them separately (Appendix, Figures A1-A3). The underlying explanations for these differences are sure to be complex and multifaceted. Nevertheless, explanations that appeal to income alone seem unlikely. Although we do observe a moderate and statistically significant correlation of 0.2 between income and meal sharing at the country level, this association explains only 4.6% of the global variation in meal sharing. Explaining the other 95.4% represents a rich opportunity for future research. While in almost every region, younger people share more meals with others, there is no consistent difference between men and women (Appendix, Figures A4-A5).

Given the considerable differences in meal sharing around the world, it is worth asking what, if anything, these differences can tell us about corresponding differences in subjective wellbeing. We start by calculating country averages for life evaluations and number of shared meals, and calculate the linear correlation between the two. We find a clear positive relationship between sharing meals and life evaluations (Appendix, Figure A6). Across countries, sharing one more meal per week is associated with an average increase of roughly 0.2 points on a scale from 0 to 10. This difference is not only statistically significant, but also practically meaningful. It is roughly equivalent to the wellbeing cost of a raise in inflation by 1.5 percentage points^37^.

**Fig. 2:**
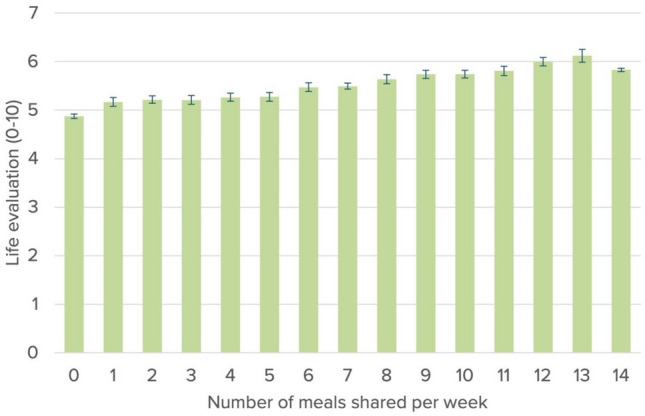
Average life evaluations by number of meals shared in the past week. Gallup World Poll, 2022–2023.

This relationship is even more apparent when analysed at the individual level. Figure 2 shows average life evaluations by the number of meals shared in the past week. In this case, our analysis is based on individual comparisons rather than country or regional averages. In general, we see an upward trend – albeit a subtle, uneven one – in average life evaluations as the number of shared meals increases. The largest difference in life evaluations is between those who eat all meals alone and those who eat one meal with someone else. People who shared just one meal in the past week have notably higher life evaluations (5.2) than those who ate all meals alone (4.9). This 0.3-point difference is statistically and practically significant. For context, it is about half as large as the decline in life evaluation associated with unemployment, which is consistently found to be one of the largest effects documented in the wellbeing literature^38–40^. We find a similar relationship when decomposing the sample by gender or age groups, and when focussing on positive and negative affect as wellbeing metrics (Appendix, Figures A7-A8).

These conditional means can, however, be contaminated by several confounders. Consider the case of income. One might imagine that eating alone is more affordable than eating with others. This seems particularly likely in high-income countries, where sharing meals with others may be more likely to take place in restaurants than at home. If so, perhaps the reason we find such a strong relationship between happiness and sharing meals is simply because people who share more meals have more money.

**Fig. 3:**
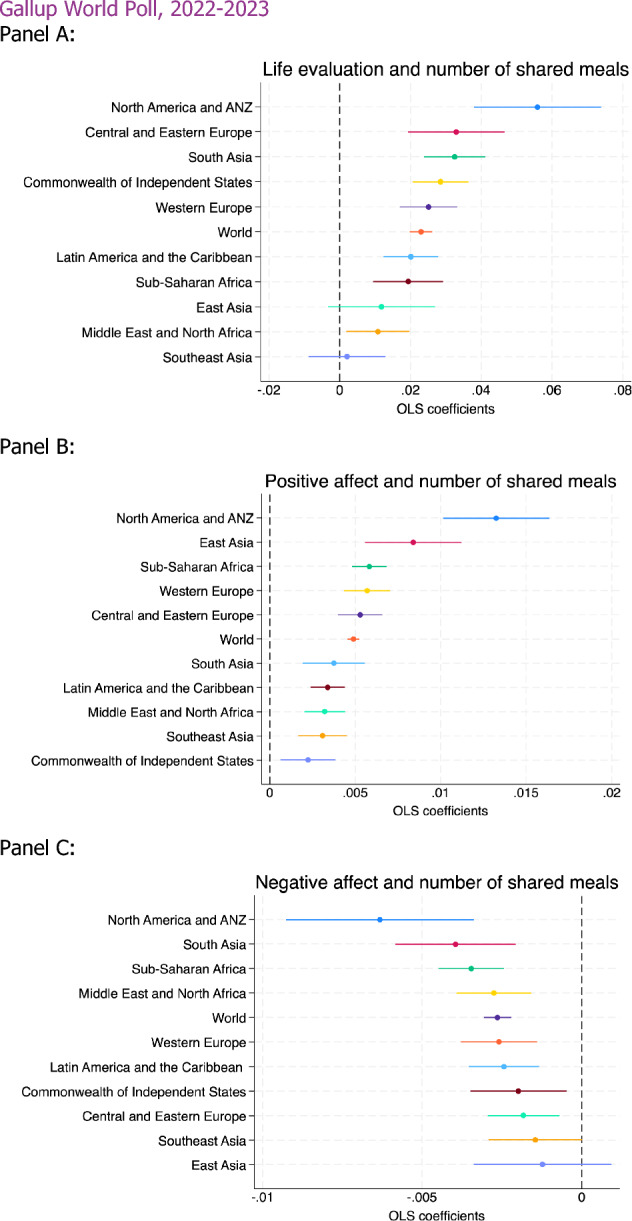
Using linear regressions to estimate the effect of sharing meals on subjective wellbeing by region. Gallup World Poll, 2022-2023. **Note:** 10 separate regressions per image. N ≈ 150,000. ANZ = Australia, and New Zealand. Controls: country fixed effects, income quintile, household size, gender, age, age-squared, education group, employment group, people’s ability to meet basic needs for food. Life evaluation is expressed on a 0-10 scale, positive and negative affect on a 0-1 scale. Data includes the 2023 sample. Predictive effect of the number of shared meals is assumed to be linear. Regressions use country-level survey weights.

To address these concerns, Fig. 3 presents the results of linear regressions which estimate the relationships between meal sharing, life evaluation, positive affect, and negative affect for all regions, while controlling for a variety of other potentially relevant factors. Specifically, we control for gender, age, age squared, binary indicators for household size, education, income quintile and employment status, as well as country fixed effects. We also control for people’s ability to meet basic needs for food, as measured by the question “Have there been times in the past 12 months where you did not have enough money to buy the food that you or your family needed?". As a result, if we continue to observe a significant relationship between sharing meals and subjective wellbeing, we can be more confident that this relationship is strong and significant in its own right, and not merely attributable to other factors.

In fact, this is precisely what we observe. Even after accounting for income, education, employment, and other key indicators, we continue to find strong and robust relationships between sharing meals and subjective wellbeing around the world. In almost all regions surveyed, sharing more meals with others proves to be highly predictive of higher life evaluations, more positive affect, and less negative affect.

We can see notable differences in the magnitude of these relationships across regions. The relationship between sharing meals and wellbeing appears to be particularly strong in North America, Australia, and New Zealand. In these countries, the differences in wellbeing between those who eat more or fewer meals alone is greater than for any other region. One potential interpretation of this result is that the importance of sharing meals with others may itself be driving the relatively high levels of meal sharing we observe in this region (Fig. 1). Indeed, sharing few or no meals could have stronger negative effects in regions where sharing many meals is the norm, and thus those that do not are outliers. However, we do not observe similar dynamics for Latin America and the Caribbean. While meal sharing is most common in this region, it does not appear particularly important for wellbeing relative to other parts of the world. At the opposite end of the spectrum, in East Asia – where meal sharing is relatively rare (Fig. 1) – we find strong links between sharing meals and negative affect, but weak links when it comes to life evaluation or positive affect.

We find similarly complex relationships between dining alone and life evaluations. Although this negative association is observed in all world regions (see the Appendix, Table A3), dining alone seems particularly detrimental in world regions where sharing meals is fairly common, but less so in regions where it is very common or very uncommon. Figure A9 in the Appendix shows the relationship between the wellbeing penalty for dining alone and the frequency of shared meals in the rest of the population. Broadly, three groups of regions appear, arranged in a U-shape pattern. First, some regions where relatively few meals are shared and those who dine alone endure a modest-to-moderate wellbeing gap (South Asia; East Asia and Sub-Saharan Africa). Second, some regions where meal sharing is fairly common and those who dine alone endure a very large wellbeing penalty (Europe; Commonwealth of Independent States; Northern America Australia and New Zealand; Middle East and North Africa). Finally, two regions where meal sharing is very common but dining alone is associated with a moderate penalty (Latin America/Caribbean and South-East Asia). When we move on to a more granular level, and estimate country-specific regression coefficients associated with dining alone, controlling for other characteristics, a similar U-shape pattern appears (see the Appendix, Figure A10).

Taken together, the relationship between how often meals are shared on average and how important meal sharing is for wellbeing is clearly not linear and not straight-forward. Examining and identifying potential explanations for these differences can provide a rich opportunity for future research.

Nevertheless, despite these differences, the overall takeaway is clear. The magnitude of these relationships may differ across regions, but their direction does not. Even after controlling for a wide variety of other factors, sharing meals continues to be strongly and consistently associated with better life evaluations, increased positive affect, and decreased negative affect.

The fact that we observe such strong and consistent links between sharing meals and wellbeing, both around the world and between individuals, is striking. But how significant are these relationships practically? Given the impressive size and scope of our dataset, it is certainly possible to find statistically significant relationships that are nonetheless relatively small, and therefore perhaps not useful for real-world applications or matters of policy.

One way to assess the practical significance of the number of shared meals is to consider how much this variable reveals about wellbeing compared to other important social indicators. To that end, we briefly return to income and employment. Decades of research has found strong and sizable links between income, unemployment, and subjective wellbeing^38–40^. The dramatic decline in life evaluation associated with unemployment in particular is one of the largest and most consistent effects to emerge from empirical wellbeing research. If we compare the informativeness of these associations with sharing meals, how do they stack up?

**Fig. 4:**
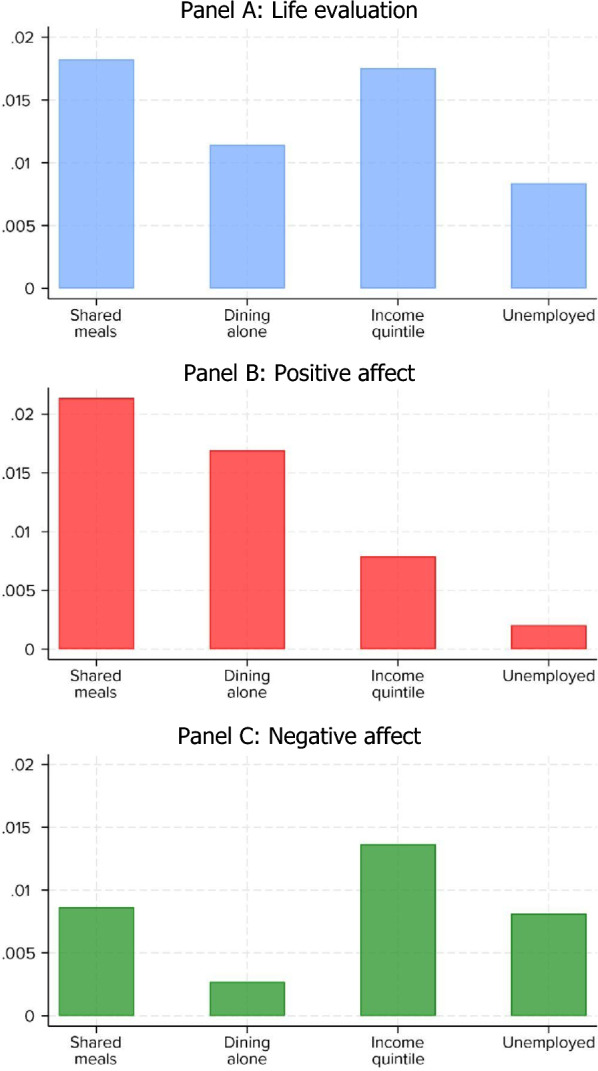
The predictive power of sharing meals, dining alone, income, and unemployment in explaining differences in subjective wellbeing around the world, Gallup World Poll, 2022–2023. **Note:** Bars represent R2 estimates from four separate regressions per image (N ≈ 150,000). No controls. Dependent variables are all expressed on a 0-100 scale and sharing meals is measured using 5 categories for comparability (0-2; 3-5; 6-8; 9-11; 12-14). Data includes the 2023 sample. The predictive effect of the number of shared meals is not assumed to be linear. Regressions use country-level survey weights.

In Fig. 4, we present the results of a series of regression analyses testing the extent to which income, unemployment, dining alone, and sharing meals explain variation in subjective wellbeing around the world. In each panel, we estimate four separate regressions in which we regress individual subjective wellbeing on the number of shared meals, dining alone (i.e., no shared meals during the past week), income quintile (within a country), and unemployment. In Panel A, we consider relationships with life evaluation, Panel B with positive affect, and Panel C with negative affect. In each case, we are measuring the extent to which differences in the independent variable of interest can explain differences in the dependent variable of interest. This is measured empirically by the R-squared value produced by each regression. By implication, the size of the bars in each figure represent the extent to which differences in e.g., shared meals can explain differences in e.g., life evaluation.

Not only do we find sharing meals and dining alone to be important predictors of wellbeing compared to income and employment, but in many cases, they seem to be even more so. That is, asking people if they shared at least one meal last week tells us more about their overall life evaluation than knowing if they are unemployed. Or relatedly, knowing how many meals someone shared last week can tell us more about their positive emotions than their income band.

Links with positive affect in particular are notable. When explaining variation in positive emotions, the extent to which people share meals with others is a more important predictor than both income and unemployment. At the same time, sharing meals does a better job explaining variation in life evaluations around the world than income or unemployment. Dining alone also emerges as a more important predictor of differences in life evaluations than unemployment, but not income. For negative affect, income continues to be a crucially important indicator, yet sharing meals and dining alone are again not far behind.

We now move on to examine whether the importance of meal sharing varies according to observable characteristics of respondents related to the frequency of social interactions. Indeed, it seems important to distinguish opportunities for social connections (having other people around) and the act of connecting (interacting with them, typically via conversations). Some recorded characteristics of the respondents, in particular the household size, can offer a hint about the respondent’s opportunity to connect. For instance, sharing seven meals per week can mean something fundamentally different for someone living alone (who must intentionally seek out social connections) versus someone in a large multi-generational household (where meals might be shared incidentally).

When decomposing the sample by household size, we find a positive relationship between the three wellbeing metrics (life evaluation, positive and negative affect) and the number of shared meals (Appendix, Figure A11). This holds true across all household sizes, including large households. Hence, we do not observe a "household size tipping point” beyond which average wellbeing declines with the number of shared meals.

Nevertheless, a more refined analysis reveals an important threshold. We focus on life evaluations and estimate a regression model with a binary variable for each household size (1,2,3,4, or 5 +) and a full interaction between each category and the number of shared meals (see Appendix, Table A4). This model allows the slope of the relationship between life evaluations and sharing meals to differ for each household size. That is, the predicted benefit of sharing one more meal is allowed to vary depending on whether someone lives alone, with one other person, etc. The results show that the slope of the relationship is different for single-households and multi-person households (t-tests, p = 0.004, p = 0.047, p = 0.001, p = 0.017), suggesting that the wellbeing benefits of an extra shared meal is larger for people living alone than with someone. Conversely, we do not find any significant difference between multi-person households of increasing sizes (t-tests, p = 0.331, p = 0.160, p = 0.215). In the Appendix (Figure A12-A15) we describe a similar type of analysis for unemployment and partnership, showing that the wellbeing gap between those with low and high opportunity of connections is reduced when sharing more meals. Taken together, these results suggest that in many regions sharing meals may be more important for those with fewer daily opportunities for social interactions.

These results clearly underscore the close connection between social eating and subjective wellbeing. Even before diving deeper into questions of causation, the fact that we observe such strong links between sharing meals, life evaluations, positive affect, and negative affect suggests that meal sharing ought to be given much more weight and attention in research and policy.

### Study 2—meal sharing over time in the United States

So far, our analysis has been based on Gallup data collected from over 150,000 survey respondents in more than 140 countries. The size and scope of this data allows us, for the first time, to compare differences in meal sharing and its relationship to subjective wellbeing for more people and more countries than ever before.

However, this survey module was only introduced in 2022, so we are unable to examine how meal sharing has changed over time. Given the close association between sharing meals and subjective wellbeing, this is an important perspective to consider as it may provide an objective yardstick by which to think about changes in subjective wellbeing over time.

To this end, this section turns to data collected by the American Time Use Survey (ATUS) in the United States from 2003 to 2023. Each year, the ATUS collects detailed information from a representative sample of roughly 12,000 Americans regarding how they spend their time on a day-to-day basis. This data has been used and referenced extensively in research, media, and policy circles. Nevertheless, to our knowledge, the extent to which Americans spend time eating alone or with others has remained relatively unexplored.

Longitudinal trends in meal sharing are particularly important when considered against the backdrop of declining social capital and connection in the United States. These trends were starkly documented by Robert Putnam in his landmark 2000 book, *Bowling Alone*^41^. Drawing on a truly expansive array of datasets, Putnam found that Americans were spending more and more time alone, while civic institutions and social organisations including religious groups, labour unions, veterans’ associations and even dinner parties were declining. These declines appeared to be occurring alongside similar declines in political participation, voting rates, trust in other people, faith in institutions, indicators of physical and mental health, rates of educational achievement, social mobility, and economic opportunity.

More recent studies have reinforced this general story of social decline in the United States and found evidence of similar trends in other countries^42,43^. One large-scale study looking at data from 1990 to 2012 in 30 European countries found that participation in social groups was falling while distrust in political institutions was rising^44^.

**Fig. 5:**
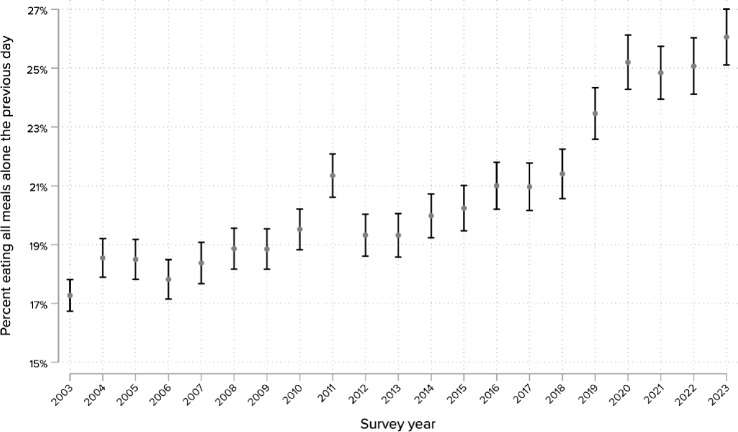
Percent dining alone in the United States, whole population. ATUS, 2003–2023. **Note: **Data from the American Time Use Survey weighted to be representative of the general population (n = 234,185). 95% confidence intervals displayed. Dining alone measured as the share of respondents in each survey year reporting eating all meals alone in the previous day.

With this context in mind, we begin our analysis by plotting the number of people dining alone in the United States over time in Fig. 5. Each dot represents the percentage of survey respondents who reported eating all of their meals alone on the previous day, encompassing over 230,000 American adults in total from 2003 to 2023. Importantly, this measure is distinct from prior sections in which we considered meal sharing over the course of an entire week using Gallup data. In this and all subsequent analyses, we also apply survey weights to our data to ensure it is representative of the general population.

The trendline is unmistakable. There has been a sharp rise in the number of Americans dining alone since 2003. Perhaps unsurprisingly, some of the highest levels of dining alone were recorded in 2019 and 2020 during the height of the COVID-19 pandemic. However, rates of dining alone were increasing long before the pandemic, and they have not come down since. In 2023, the most recent year for which data is available, rates of dining alone in the United States were even higher than they were during the pandemic. Roughly 1 in 4 American adults (26%) reported eating all of their meals alone in the previous day – representing an overall increase of more than 50% since 2003. In the Appendix, we present a series of robustness checks in Figure B1 in which we expand our definition of dining to include cooking and food preparation, and consider the percent of total number of meals eaten alone. In each case, results point in the same direction.

The clear takeaway is that, in one of the most reliable, reputable, and widely used time use datasets in the world, we find clear and consistent evidence that, with every passing year, Americans are spending more and more time dining alone.

**Fig. 6:**
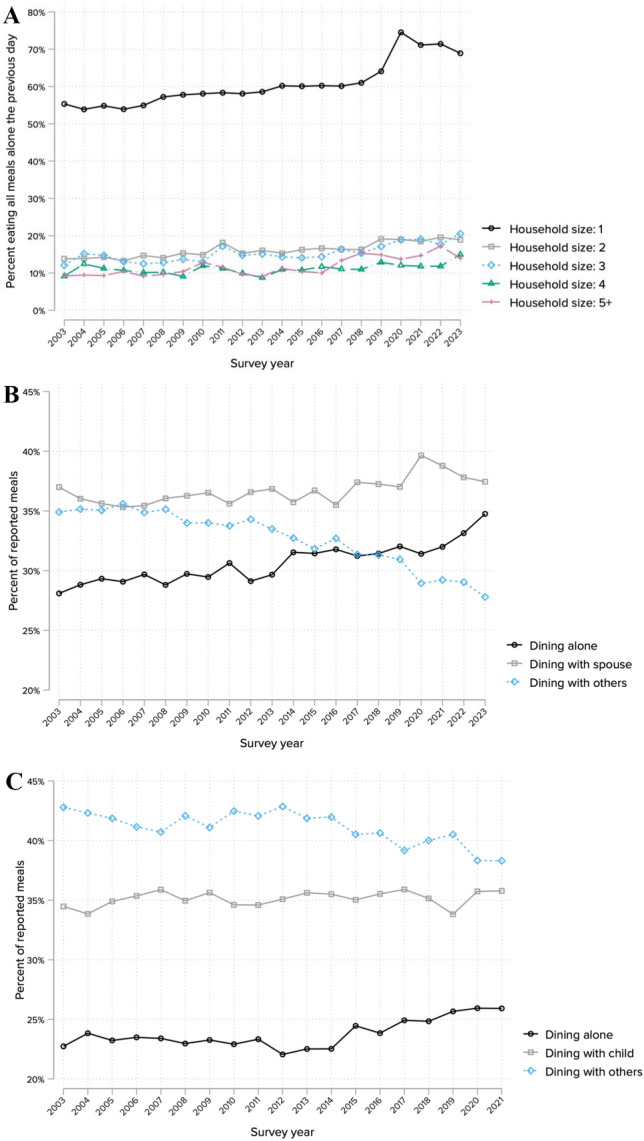
Dining alone in the United States, by household composition. Panel A: Dining alone in the United States, by household size. ATUS, 2003–2023. **Note:** Data from the American Time Use Survey weighted to be representative of the general population (n = 234,143). Dining alone measured as the share of respondents in each survey year reporting eating all meals alone in the previous day. Panel B: Dining alone in the United States among married adults. ATUS, 2003–2023. **Note:** Data from the American Time Use Survey weighted to be representative of the general population. Sample limited to married adults (n = 122,066). Meal sharing measured as the percent of reported meals eaten alone or with others in the previous day. Panel C: Dining alone in the United States among parents living with children. ATUS, 2003–2023.** Note: **Data from the American Time Use Survey weighted to be representative of the general population. Sample limited to parents living with children (n = 83,611). Meal sharing measured as the percent of reported meals eaten alone or with others in the previous day.

These trends also appear to be broadly consistent across demographic groups. One of the most obvious potential explanations for the rise in dining alone is the associated rise in living alone. Although people who live alone are considerably more likely to eat alone (Fig. 6, Panel A), and more Americans live alone today than two decades ago (Appendix Figure B2), regression analyses estimate that the rise in living alone accounts for 17% of the increase in dining alone (Appendix Figure B3). This is estimated as the difference in R^2^ values of two regressions with year dummies estimating the change in dining alone over time with and without controlling for the increase in living alone. Other factors are clearly at play, as significant increases remain even after controlling for variables such as household size, age, gender, and income. Indeed, the largest increases in dining alone since 2003 occurred among those living with others, rising from 12 to 18%, a 50% increase. Among those living alone, the increase was smaller, from 55 to 69%, a 25% rise.

We find similar increases in dining alone when we limit our sample to married adults living together and parents living with children. However, in both cases, these rises appear largely attributable to declines in sharing meals with those who live outside the household. That is, married adults living together (Fig. 6, Panel B) and parents living with children (Fig. 6, Panel C) appear just as likely to eat together as they were in 2003. However, both groups have become considerably less likely to share meals with non-household members.

Another important factor to consider in explaining the rise in dining alone is the expansion of remote work in the United States. In the ATUS, we find that the percentage of respondents who report working from home at least some of the time had been steadily rising before 2020, and increased considerably during the course of the pandemic (Appendix Figure B4). However, this too does not appear to explain the rise in dining alone (Appendix Figure B3). Although working from home has become increasingly common, remote workers still account for a relatively small proportion of the American workforce. Even more importantly, the relationship between working from home and dining alone appears to be ambiguous. Workers who work from home may even become more likely to share meals if they live with other people than they would be eating at their workplaces. In fact, we find that employed adults who do and do not report working from home are just as likely to report dining alone, and that rates of dining alone have increased along almost identical trajectories since 2003 for both groups (Appendix Figure B5).

**Fig. 7:**
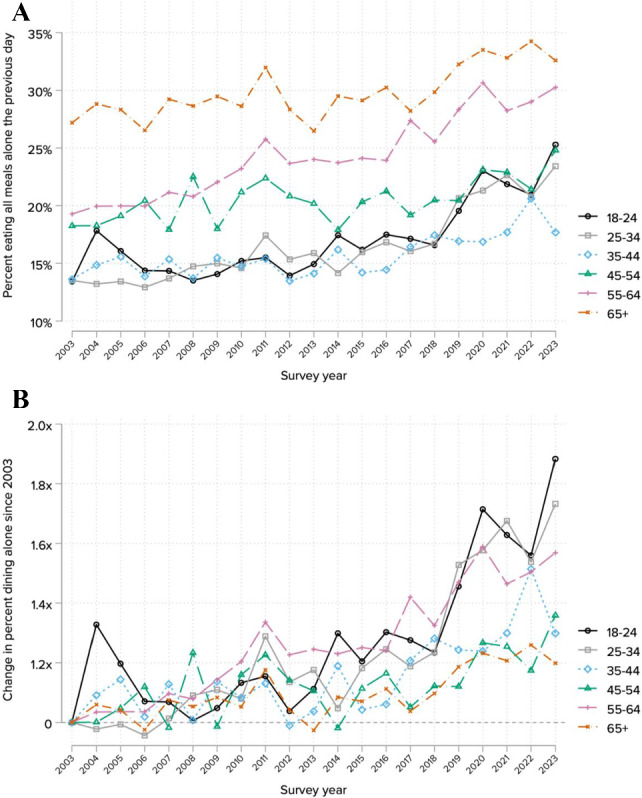
Dining alone in the United States by age. Panel A: Rates of dining alone in the United States, by age. ATUS, 2003–2023. **Note:** Data from the American Time Use Survey weighted to be representative of the general population (n = 234,185). Dining alone measured as the share of respondents in each survey year reporting eating all meals alone in the previous day. Panel B: Changes in dining alone in the United States, by age. ATUS, 2003–2023.** Note:** Data from the American Time Use Survey weighted to be representative of the general population (n = 234,185). Dining alone measured as the share of respondents in each survey year reporting eating all meals alone in the previous day.

Turning to gender, while men eat alone more often than women do, the increasing trend in dining alone is similar across genders (Appendix Figure B6). However, age group analysis reveals distinct patterns. Figure 7, Panel A, shows that older adults (65 +) consistently report higher rates of dining alone compared to younger cohorts, aligning with the global trends reported in Study 1. The fact that older people are less likely to share meals than younger counterparts may be unsurprising given that older individuals are both more likely to live in smaller households and more likely to have lost friends and family members to death and disease. Nevertheless, when we consider trends in meal sharing over time, the temporal analysis shows that young adults are disproportionately driving the upward trend in dining alone.

This is apparent in Fig. 7, Panel B, where we normalise rates of dining alone for all age groups to their 2003 levels, and then plot overall changes for each cohort in the years since. The first thing to note is that levels of dining alone have clearly increased for every age group since 2003. This is true even among older cohorts who were already much more likely to dine alone 20 years ago. Nevertheless, the largest and most dramatic changes are for those under the age of 35. The extent to which 18 to 24-year-olds report eating all of their meals alone in the previous day has increased by about 90% in two decades. We observe a similarly dramatic increase for 25 to 34-year-olds.

Such sizable increases in rates of dining alone among young adults in the US clearly and urgently warrant further research and policy attention. We are not the first to document concerning levels of isolation among young people. In fact, the sharpest rises in dining alone among young people that have occurred since 2011 seem to be closely aligned with the timing of well-documented associated declines in mental health and increases in despair^21,45,46^. This suggests that the causes of both increases in dining alone and increases in mental health concerns are likely to be deep rooted and long term. Nevertheless, many of the existing explanations emanating from public discourse and the academic literature are somewhat unsatisfying in the present context.

Most notably, the rise of smartphones and social media are often credited with observable declines in young people’s wellbeing. However, when it comes to dining alone, the timelines do not line up as neatly as one might expect. With the launch of Facebook in 2004 and the introduction of the iPhone in 2007, one might expect that the sharpest increases in rates of dining alone would emerge around these times. Instead, we observe a relatively steady and consistent rise in young people dining alone from 2003 to 2015, followed by increasingly steeper inclines in the years since.

Another common explanation for (or at least contributor to) the decline in young people’s mental health has been the COVID-19 pandemic. Indeed, we do see some of the highest recorded rates of dining alone during the course of the pandemic in 2020 and 2021. However, rates of dining alone were increasing long before the pandemic began to spread in the United States. During the course of the pandemic, rates of dining alone among young people even appear to have declined slightly – potentially reflecting more meals eaten at home with family members. If the pandemic was the whole story, we may also expect rates of dining alone among young adults to have declined in more recent years. In fact, we observe the highest levels of dining alone among those under 35 in 2023. While we certainly do not reject the notion that COVID-19 may have contributed to higher levels of isolation among young adults, the timing suggests that it has not been the primary driver of increases in dining alone.

For the remainder of this section, we turn from the overall levels and trends in meal sharing, to the relationship between meal sharing and subjective wellbeing. In 2010, 2012, 2013, and 2021, the American Time Use Survey included a special wellbeing module. In the remainder of the Study, we focus on these years in particular. In the wellbeing module, all respondents were asked about their overall satisfaction with life—using the Cantril Ladder—and their daily emotions—using a partial Day Reconstruction Method, where they were asked how often and to what extent they felt happy, sad, in pain, tired, and stressed during three episodes that occurred during the previous day.

**Fig. 8:**
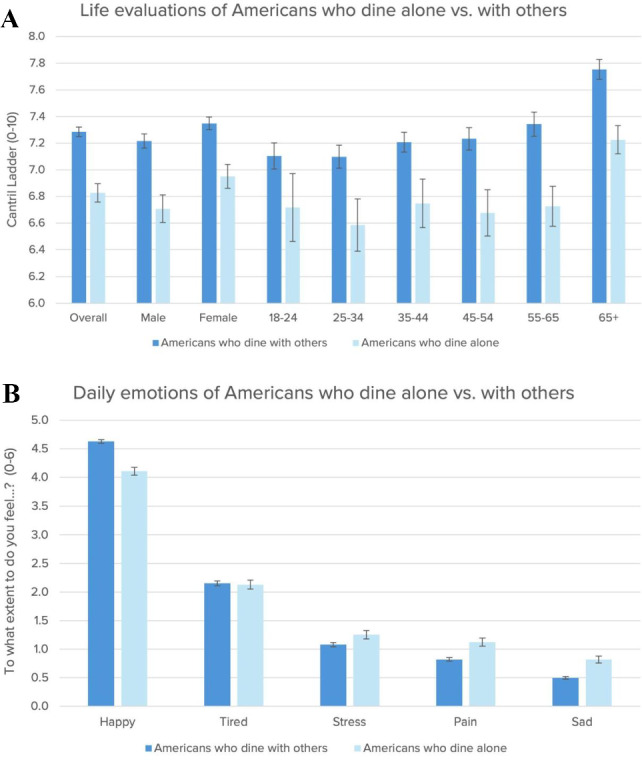
Subjective wellbeing of Americans who ate all meals alone the previous day compared to those who shared meals. Panel A: Life evaluations. ATUS (2010, 2012, 2013, 2021). Panel B: Daily emotions. ATUS (2010, 2012, 2013, 2021). **Note: **Bars represent average levels of life satisfaction (Panel A) and daily affect (Panel B) reported by those who ate all meals alone the previous day vs. those who ate at least one meal with someone else. Data weighted to be representative of the general population. (n =28,811).

In Fig. 8, Panel A, we use this data to compare average differences in life evaluation between Americans who report eating all of their meals alone in the previous day relative to those who shared meals. We find that Americans who dine alone report life evaluations that are, on average, 0.5 points lower than those who dine with others. Similarly large differences in life evaluations are observable when we split our sample by age cohorts and gender**.** Dining alone is strongly associated with substantial differences in subjective wellbeing for both men and women, young and old alike.

In Fig. 8, Panel B, we present analogous results for positive and negative affect. Again, we split our sample by Americans who report eating all meals alone the previous day compared to those who ate at least one meal with someone else and plot the average levels of daily emotions for each group. In line with prior results, we observe notable differences in emotions between those who share meals and those who dine alone. Differences in happiness, sadness, and pain are particularly large, though we also find slight differences in self-reported levels of stress. In each case, Americans who eat more meals with others report higher levels of positive affect and lower levels of negative affect than those who dine alone. We find no significant differences for tiredness.

In Tables B1-B2 of the Appendix, we conduct a series of additional robustness checks in which we estimate the size and strength of the relationship between dining alone and subjective wellbeing using linear regressions. In every instance, we find that differences in life evaluations, positive affect, and negative affect between Americans who dine alone and Americans who share meals are statistically significant at a 99% confidence level. This is true even after controlling for age, sex, geographic location, marital status, and race. In an echo of earlier results, relationships with positive affect are strongest. In Table B3, using standardized measures of all wellbeing outcomes, we find that gaps in happiness in particular are larger than those estimated for life evaluations, or any other affect measure under consideration. Finally, we also find that differences in wellbeing between those who share meals and dine alone do not appear to vary by marital status, household composition, employment status, or remote working arrangements (Appendix, Table B4). This stands in contrast to the global trend documented in Study 1, which showed that differences in wellbeing between those with varying opportunities for daily social interactions shrinks as people share more meals (Appendix, Figure A12). However, it is in line with the specific results for North America where such convergence is not observed (Figures A13, A14, A15).

Taken together, our results in Study 2 point to concerning declines in how often Americans share meals with each other. It is again worth noting that, unlike other social indicators such as loneliness or depression, the relatively objective nature of sharing meals makes it a uniquely reliable metric by which to compare differences over time. The fact that fewer Americans report sharing meals with others today than ever before is particularly concerning given the close relationship between meal sharing and subjective wellbeing – a relationship that we have now confirmed in two separate large-scale nationally and internationally representative datasets.

## Discussion

In this paper, we have presented new evidence on the global variation in meal sharing and what it implies for subjective wellbeing and social connections.

Overall, we find stark differences in meal sharing around the world. These differences prove to be closely tied to age – on average, younger people share more meals with others than older adults – but mostly unrelated to gender. Global differences in meal sharing are also not fully explained by regional or individual characteristics such as income, employment, or household size. When we examine the link between sharing meals and subjective wellbeing, we find that individuals who share more meals with others generally report higher levels of life satisfaction, lower levels of negative affect, and especially higher levels of positive affect. In the United States, rates of meal sharing appear to be in stark decline, while rates of dining alone are on the rise. This is true for both genders and all ages, but especially so for young adults.

Overall, the strong and robust relationships between sharing meals, wellbeing, and social connections that we document in this paper cry out for future research and exploration. Unlike most indicators of social relationships, and all indicators of subjective wellbeing, the number of meals shared with others is relatively objective and directly comparable across individuals, between countries, and over time. This feature makes sharing meals (and its counterpart, dining alone) uniquely valuable and well-positioned to reveal new insights into the nature and dynamics of human wellbeing.

Our analysis also has some limitations. First, and most importantly, we are unable to address the key question of causation. There is already considerable evidence that social connections contribute to greater wellbeing^4,6,47,48^and early suggestive evidence that sharing meals with others may promote social connection^34–36,41^. However, more research is clearly warranted. Second, in the analyses of the Gallup World Poll, we are unable to properly control for the fact that some respondents might skip either lunch or dinner. This element might be particularly relevant in countries where many citizens are exposed to food insecurity. Third, although our two studies converge in highlighting the association between meal sharing and wellbeing, they do not delve deeply into the mechanisms driving this relationship. Factors such as the quality of social interactions, the type of meal, or other confounding variables (e.g., work schedules) may play a significant role but are not explored.

As a final point, it is worth noting the possible policy implications of these findings. While researchers and policymakers have long lamented declines in social connectedness across modern societies, isolating the key contributing factors and, even more importantly, identifying solutions has proven frustratingly difficult. If sharing meals proves to be just as important for promoting social ties and subjective wellbeing as the evidence in this paper suggests, it may serve as a uniquely valuable, actionable, and cost-effective policy tool by which to facilitate and promote social welfare.

## Methods

Our methods were carried out in accordance with all relevant guidelines and regulations. Since this study relied on secondary observational data, formal ethical approval was not sought for this study.

Our analyses use univariate and multivariate regression models, in addition to summary statistics and data visualizations of aggregate statistics including means and longitudinal time trends. All regressions reported in the studies are estimated by Ordinary Least Squares (OLS), unless indicated differently. Survey weights are used to ensure that the samples are representative of the country’s population. More details are reported below.

### Study 1—data

Study 1 uses data from the Gallup World Poll (GWP). The GWP is conducted annually across more than 150 countries, captures data from nationally representative samples of residents, covering over 99% of the global adult population. Each year, approximately 1,000 individuals are surveyed per country or region using random sampling techniques. Surveys were carried out in accordance with the guidelines and regulations reported in the Gallup World Poll “Methodology Handbook” and the “Declaration of Helsinki". An ethical board has reviewed the 2022 and 2023 World Poll questionnaires, and participants have given informed consent about the use of their data. Data is available for purchase from Gallup.inc.

### Study 1—measures

The survey employs a standardized set of core questions, translated into the predominant languages of each country, to ensure consistent and comparable results. In 2022, participants from over 140 countries and territories were asked about their social dining habits with the following questions: "Thinking about the last 7 days, on how many days did you eat lunch with someone you know?" and "Thinking about the last 7 days, on how many days did you eat dinner with someone you know?". In 2023, Gallup introduced a slight change in wording and asked respondents from 17 countries: “Thinking about the past 7 days, on how many days did you eat LUNCH/DINNER with someone you know; including family, friends, or anyone else you know.” Countries surveyed in 2023 include: Albania, Cambodia, Czechia, Denmark, Egypt, El Salvador, Germany, Japan, Kazakhstan, Malaysia, Moldova, Paraguay, Sénégal, Sierra Leone, Togo, Tunisia, and Zambia. In the Gallup World Poll, life evaluation is measured using Cantril’s "ladder of life" scale^49^, i.e.: "Please imagine a ladder, with steps numbered from 0 at the bottom to 10 at the top. The top of the ladder represents the best possible life for you, and the bottom of the ladder represents the worst possible life for you. On which step of the ladder would you say you personally feel you stand at this time?". Positive and negative affect are measured based on the measures used in the World Happiness Report^50^. Positive affect is defined as the average of three positive affect measures in GWP: laugh, enjoyment and doing interesting things. These measures are the responses yes/no to the following three questions, respectively: “Did you smile or laugh a lot yesterday?”, and “Did you experience the following feelings during A LOT OF THE DAY yesterday? How about Enjoyment?”, “Did you learn or do something interesting yesterday?”. Negative affect is given by the average of individual yes/no answers about experiencing worry, sadness, and anger the day before. The three questions are, respectively: “Did you experience the following feelings during A LOT OF THE DAY yesterday? How about Worry?”, “Did you experience the following feelings during A LOT OF THE DAY yesterday? How about Sadness?”, and “Did you experience the following feelings during A LOT OF THE DAY yesterday? How about Anger?”.

### Study 1—statistical analysis

We conduct both descriptive and inferential analyses. All statistical tests are two-sided. Statistical significance is defined as p-value < 0.05.

First, we compute weighted descriptive statistics, including country-level averages of meal sharing (Fig. 1), and mean life evaluations conditional on the number of meals shared (Fig. 2).

Second, we analyze individual-level relationships between meal sharing and subjective wellbeing using linear regression models. Unless otherwise specified, models are estimated using OLS with country-level survey weights., and multivariate regressions control for country fixed effects and the following set of sociodemographic and economic covariates: gender, age, age squared, household size (1; 2; 3; 4; 5 or more), education group (elementary education or less; secondary education; college), income quintile (within country), and employment status (full-time employee; full-time self-employed; employed part time; underemployed; unemployed; out of workforce). In addition, we control for respondents’ ability to meet basic food needs (yes/no), based on whether they report having lacked money to buy food in the past 12 months. To examine heterogeneity, we estimate models separately by world region (Fig. 3), and conduct subgroup analyses by gender, age, and household size. In particular, we estimate interaction models between household size categories and the number of shared meals to allow the association with life evaluation to vary across household types. Differences in coefficients across groups are assessed using t-tests.

Finally, to compare the relative explanatory power of different predictors (meal sharing, dining alone, income quintile, and unemployment status), we estimate separate bivariate OLS regression models and report the corresponding R^2^ (Fig. 4). In these specifications, the number of shared meals is grouped into five categories (0–2; 3–5; 6–8; 9–11; 12–14), for comparability with income quintiles (which also appears in five catergories).

### Study 2—data

Study 2 uses data from the American Time Use Survey (ATUS). ATUS is conducted annually by the U.S. Census Bureau since 2003, is a nationally representative survey designed to measure how people allocate their time across various activities, such as work, leisure, childcare, and household tasks, using a time diary format. More information is available here: https://www.ncbi.nlm.nih.gov/books/NBK179213/. Data is publicly available for research purposes free of charge.

### Study 2—measures

While there is no direct measure of meal sharing or dining alone in the American Time Use Survey, we construct both variables by limiting our analysis to activity periods in which respondents report their primary activity to be eating and drinking, either with others or by themselves. We also consider all meals eaten with others as instances of meal sharing. That is, our primary analyses do not distinguish between meals shared with e.g., partners, children, other family members, friends, housemates, etc. In some analyses, we consider individual meals as our primary variable of interest, while in others we consider all meals eaten alone in the previous day as our primary indicator of dining alone. In a variety of robustness checks presented in the Appendix, we also expand our definition of meals to include time spent on activities related to eating and drinking – e.g., waiting in line for food orders, time spent on food preparation and cleaning, etc. These inclusions do not substantially alter our results. A Well-Being Module, funded by the National Institute on Aging, was also integrated into the ATUS in 2010, 2012, 2013, and 2021. This module collected self-reported data on emotional experiences during three randomly chosen episodes of the day. The ATUS dataset comprises over 1,307,107 episodes relating to eating, food preparation, and cooking from 239,272 unique respondents. The Well-Being Module contains 46,767 of these episodes from 25,169 unique respondents. Participants reported their feelings during selected activities using a partial “Day Reconstruction Method", where they were asked to recall their emotions for specific moments of the previous day^51^. Respondents rated their experiences of happiness, fatigue, stress, sadness, and pain on a scale from 0 (not experienced at all) to 6 (very intense). The wording was the following: “between [the start time of the activity episode] and [the time at which the episode ended] yesterday, you said you were doing [stated activity]. The next set of questions asks how you felt during this particular time. Please use a scale from 0 to 6, where a 0 means you did not experience this feeling at all and a 6 means the feeling was very strong.” for the ratings referenced the exact times and activities for each episode, ensuring contextually relevant responses.

### Study 2—statistical analysis

We conduct both descriptive and regression-based analyses. All statistical tests are two-sided. Statistical significance is defined as p-value < 0.05.

First, using ATUS sampling weights, we compute annual estimates of the share of respondents who report eating all meals alone on the diary day, and examine trends over time from 2003 to 2023. These estimates are presented graphically with 95% confidence intervals (Fig. 5). We further examine trends across demographic subgroups, including household size, marital status, parental status, employment characteristics, gender, and age groups (Figs. 6 and 7).

For analyses of subjective wellbeing, we compare weighted mean levels of life evaluation and daily affect between respondents who report eating all meals alone and those who share at least one meal, with corresponding 95% confidence intervals (Fig. 8).

We also estimate linear regression models using OLS to assess the association between eating all meals alone and subjective wellbeing outcomes. These models control for age, sex, race (white, black, and other), marital status (married, widowed, divorced, separated, and never married), and include county fixed effects. Finally, we examine heterogeneity in these associations using subgroup-specific models and interaction terms across groups defined by marital status, household size, employment status, and remote working status.

## Supplementary Information

Supplementary Information 1.

## Data Availability

The microdata used for Study 1 are available from Gallup, but restrictions apply to the availability of these data, which were used under license for the current study, and so are not publicly available. Data requests should be addressed to the corresponding author. The microdata used for Study 2 are publicly available and free of charge for research purposes. They are provided by the U.S. Bureau of Labor Statistics: https://www.bls.gov/tus/modules/wbdatafiles.htm
