# Supplementary material for: Sharing meals is associated with greater wellbeing
Source: Sci Rep. 2026 Apr 22;16:18741. doi: 10.1038/s41598-026-46771-9 (PMC13272623; doi:10.1038/s41598-026-46771-9)
Supplement: Supplementary file 1 — Supplementary Information 1. [file 41598_2026_46771_MOESM1_ESM.docx]

# **Appendix**

# **Study 1 - Figures**

**Figure A1:**
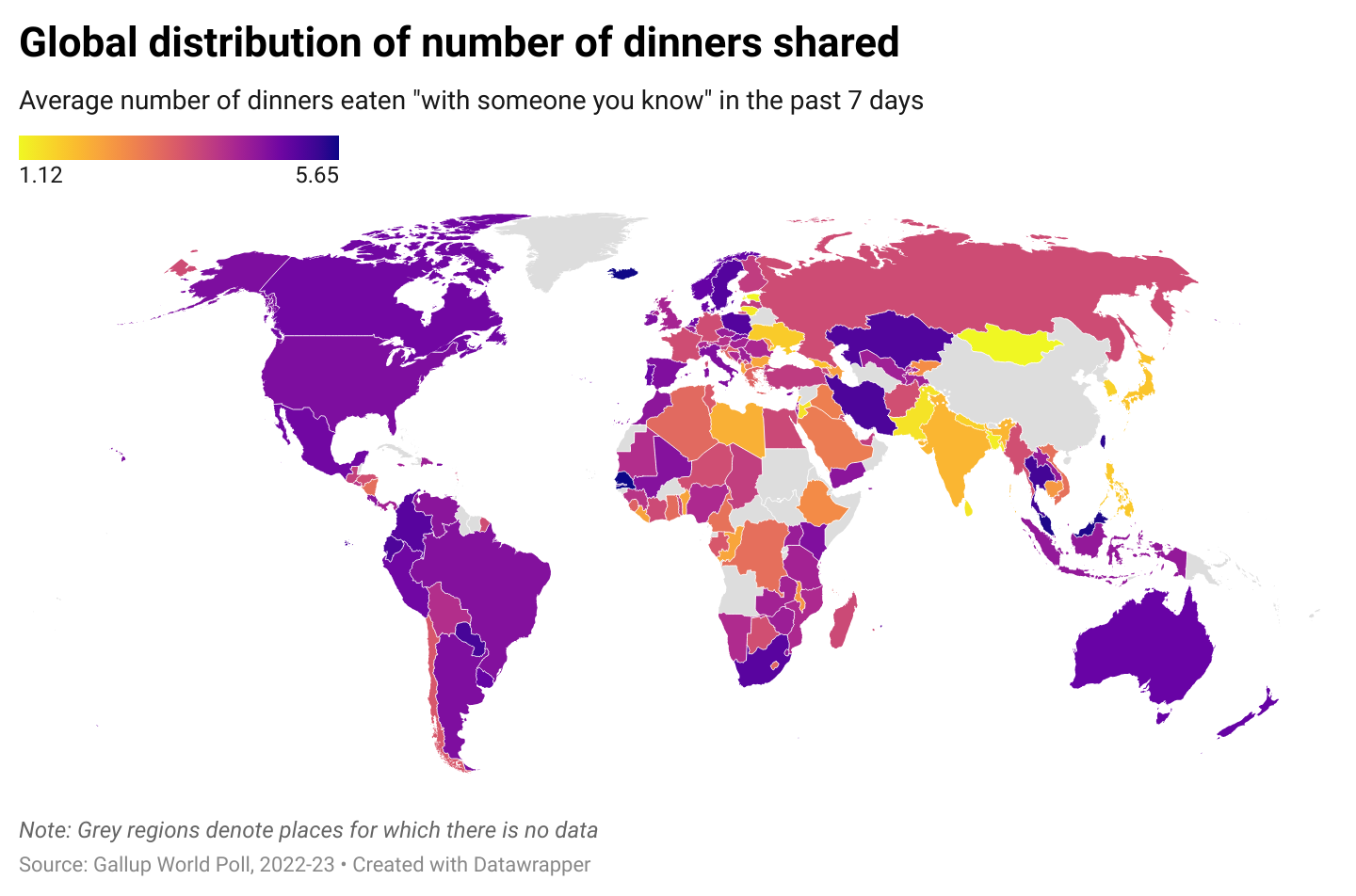


**Figure A2:**
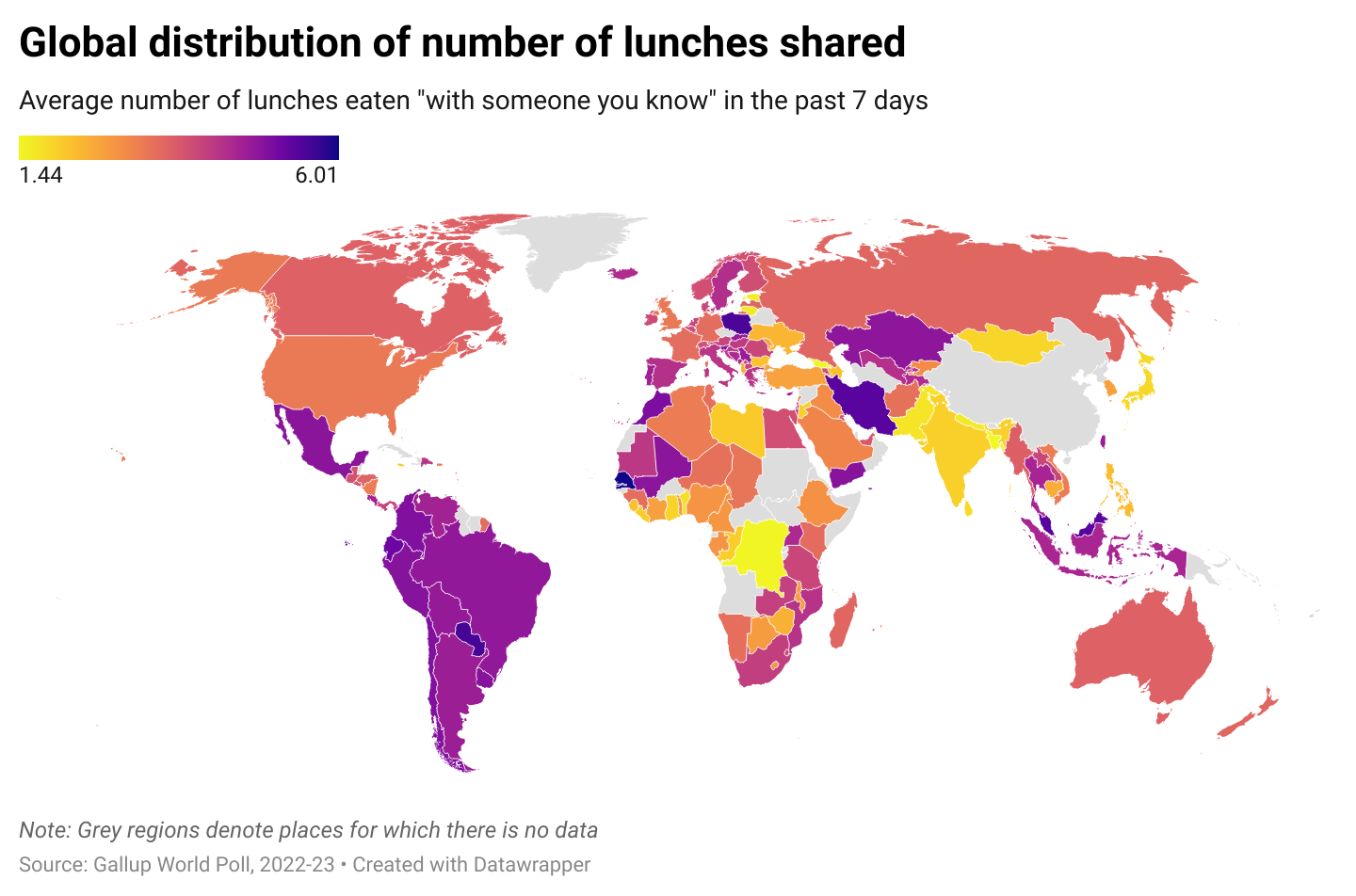


| **Figure A3: Meal sharing by region**  Gallup World Poll, 2022-2023  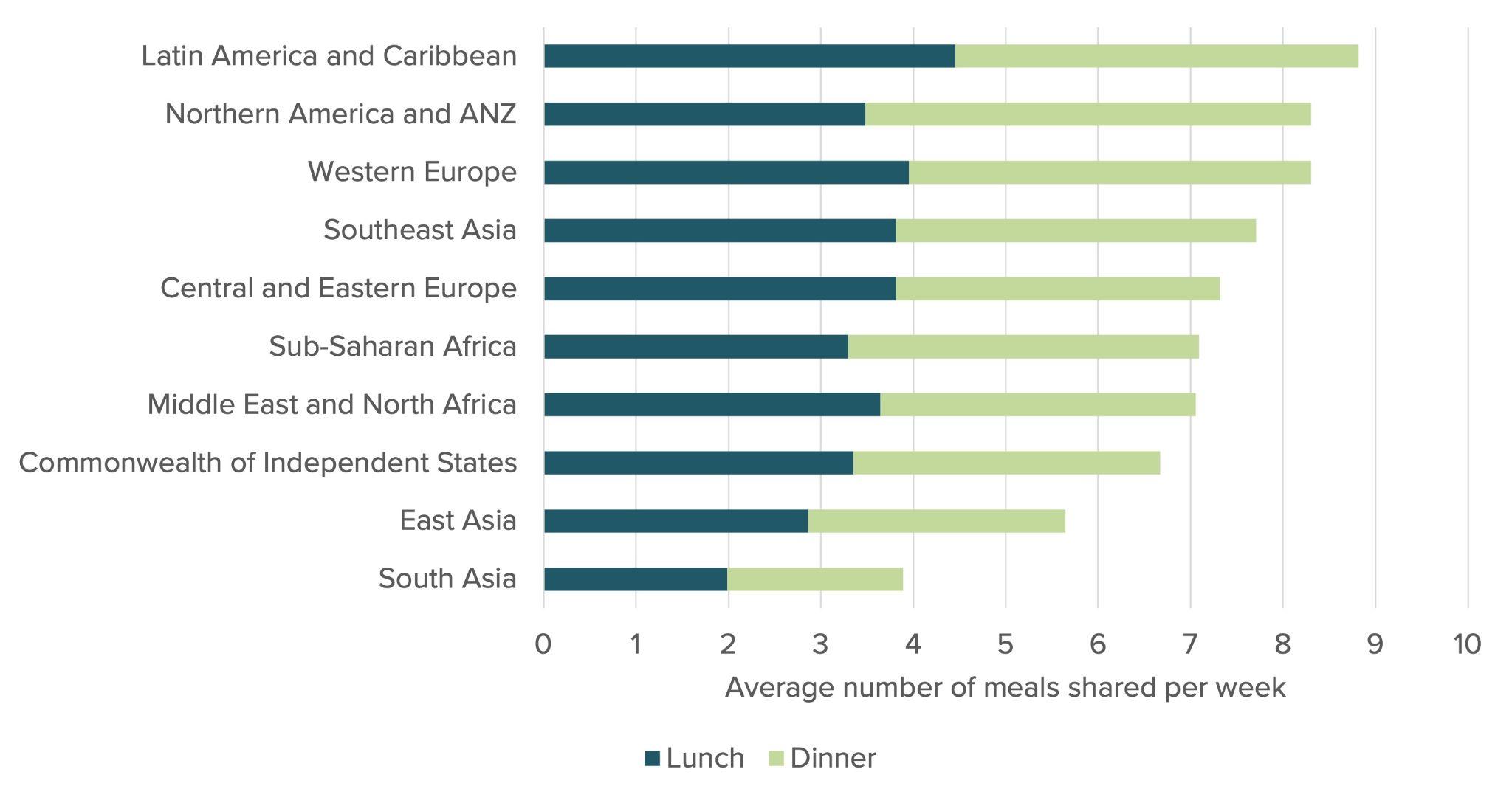  **Figure A4: Meal sharing by region and gender** Gallup World Poll (2022-2023) |
| --- |
| 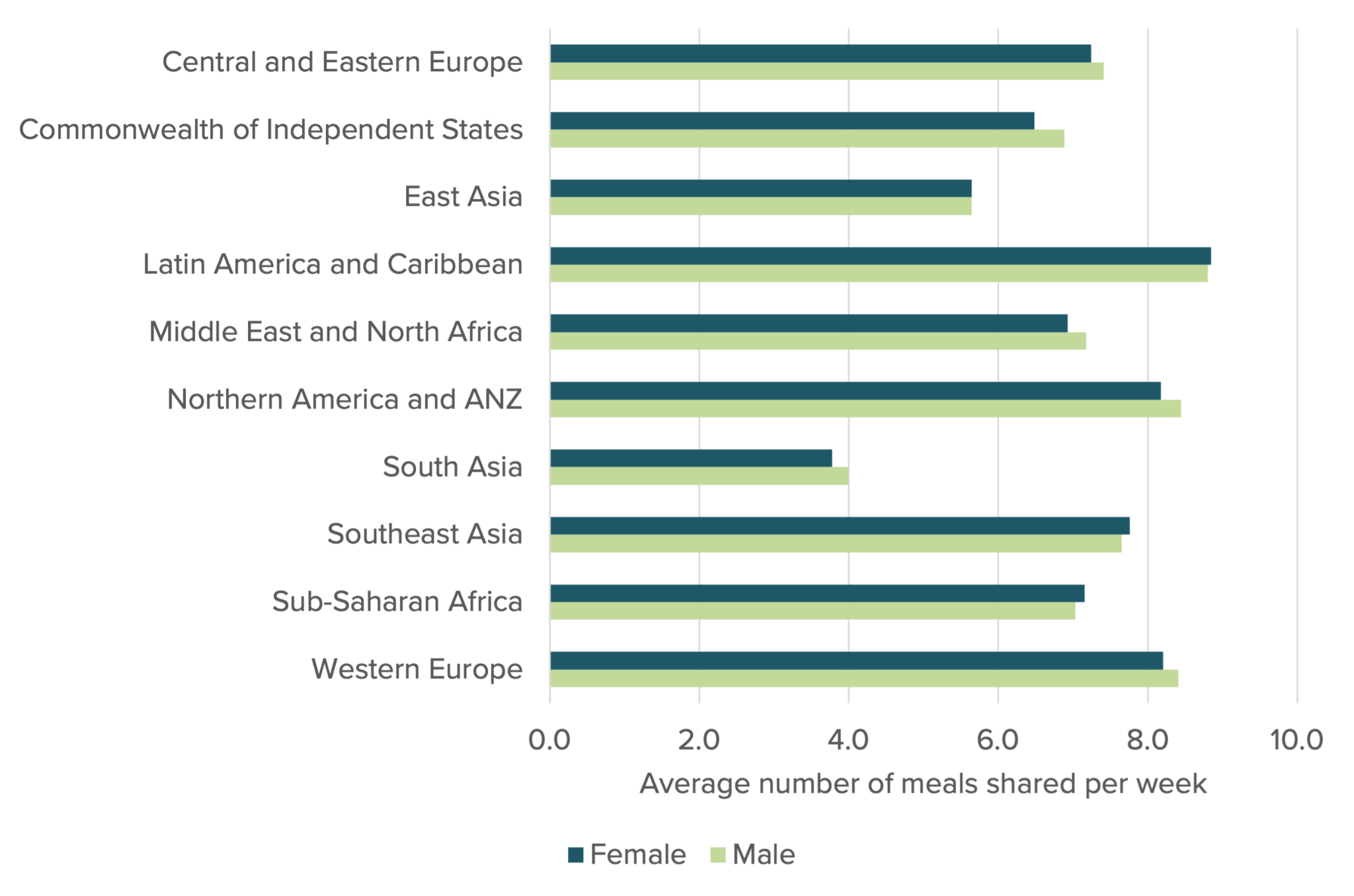 |
|  |

**Figure A5: Meal sharing by region and age**Gallup World Poll, 2022-2023

| 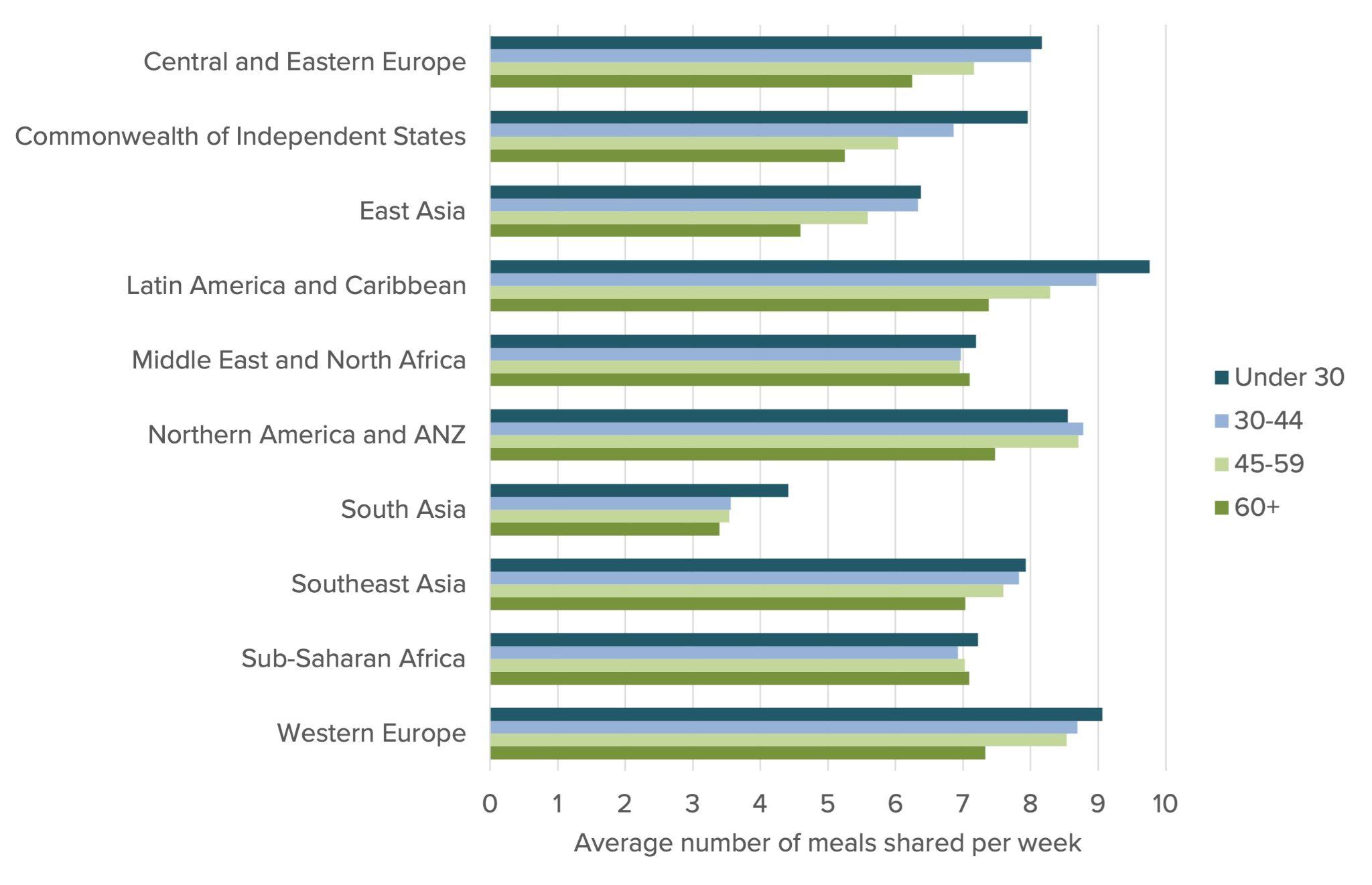 |
| --- |

**Figure A6: Meal sharing and life evaluations around the world**Gallup World Poll, 2022-2023


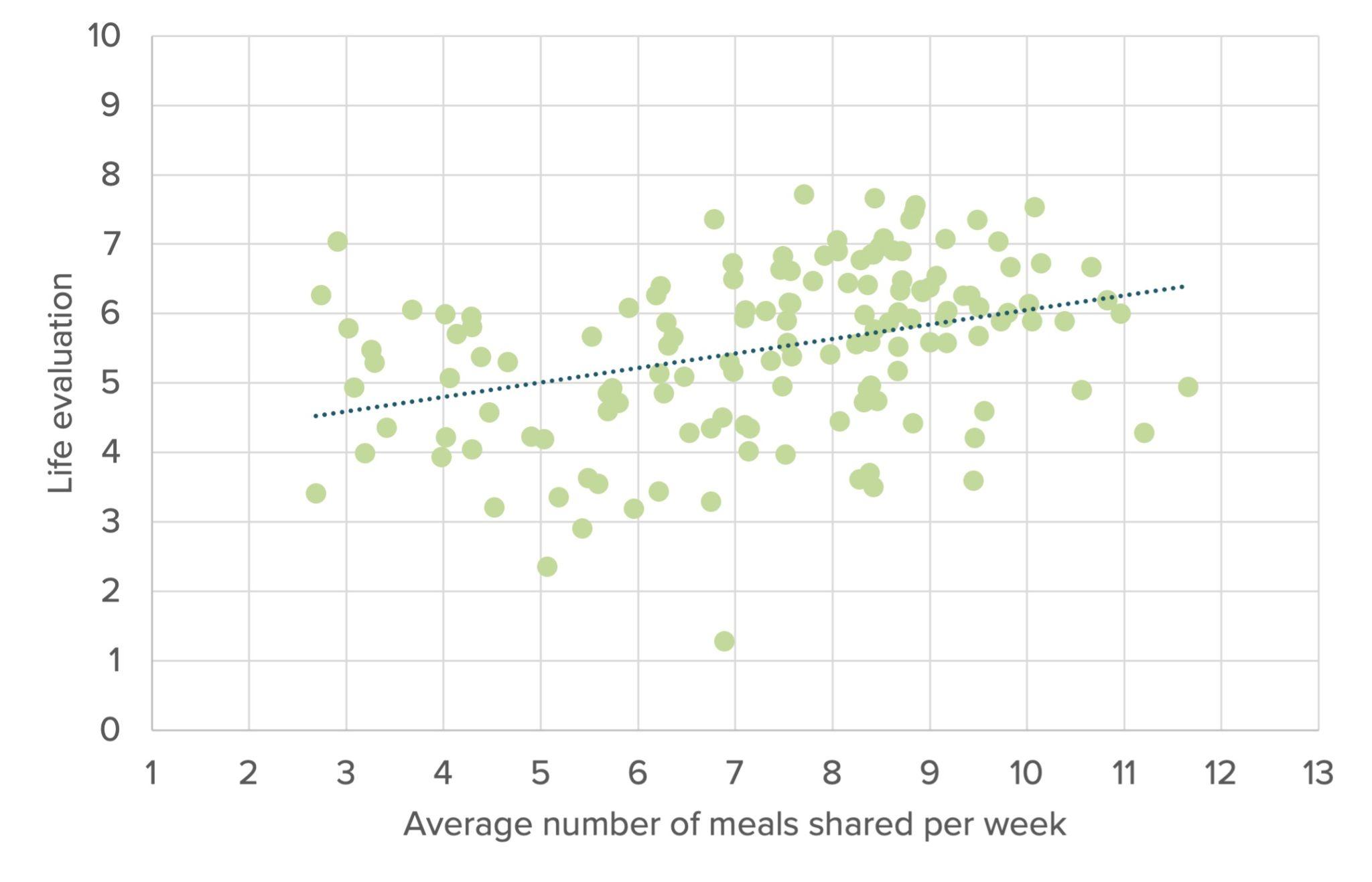


**Figure A7: Shared meals and subjective wellbeing by gender**Gallup World Poll, 2022-2023

**
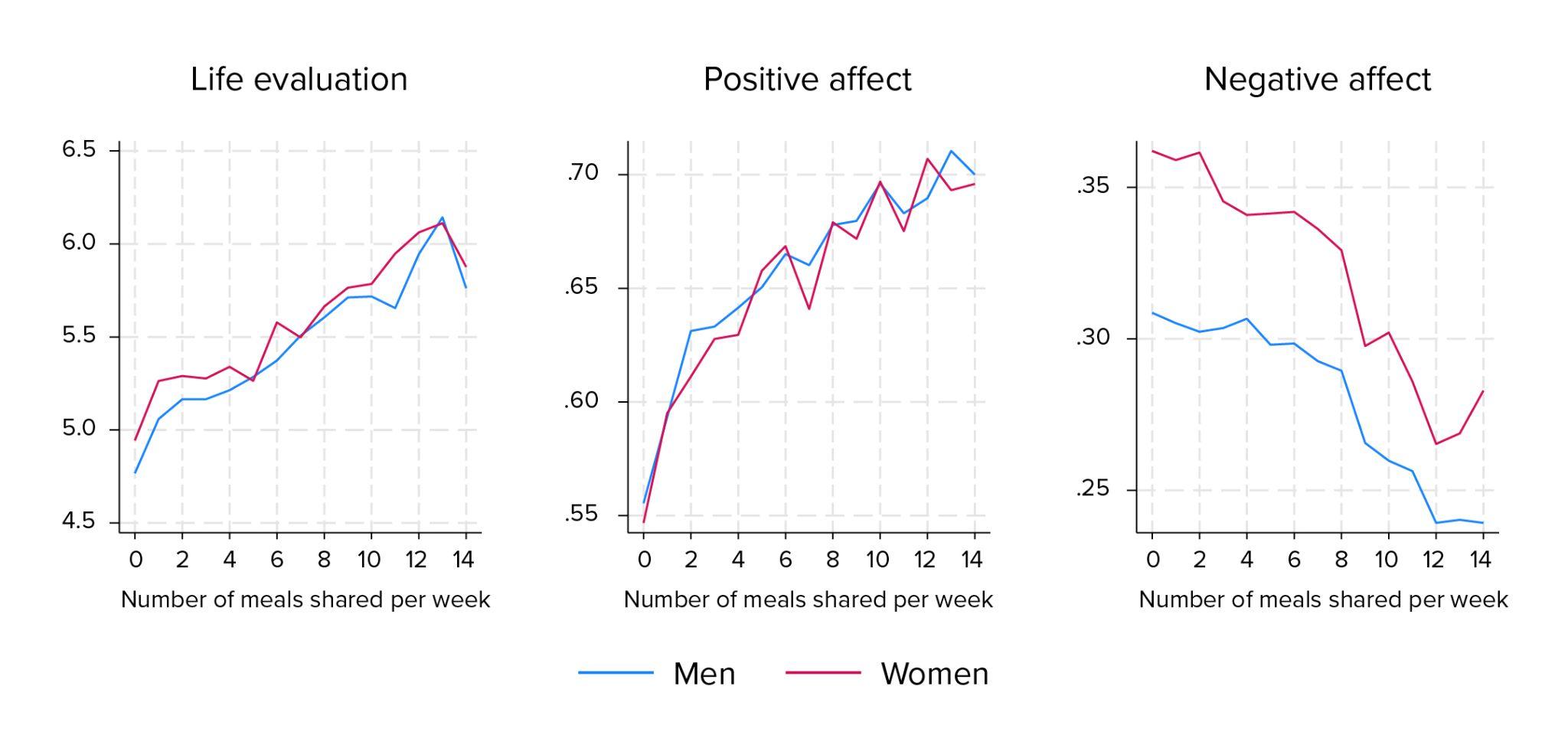
**

**Figure A8: Relationships between sharing meals, life evaluation, positive affect, and negative affect around the world by age**

Gallup World Poll (2022-2023)


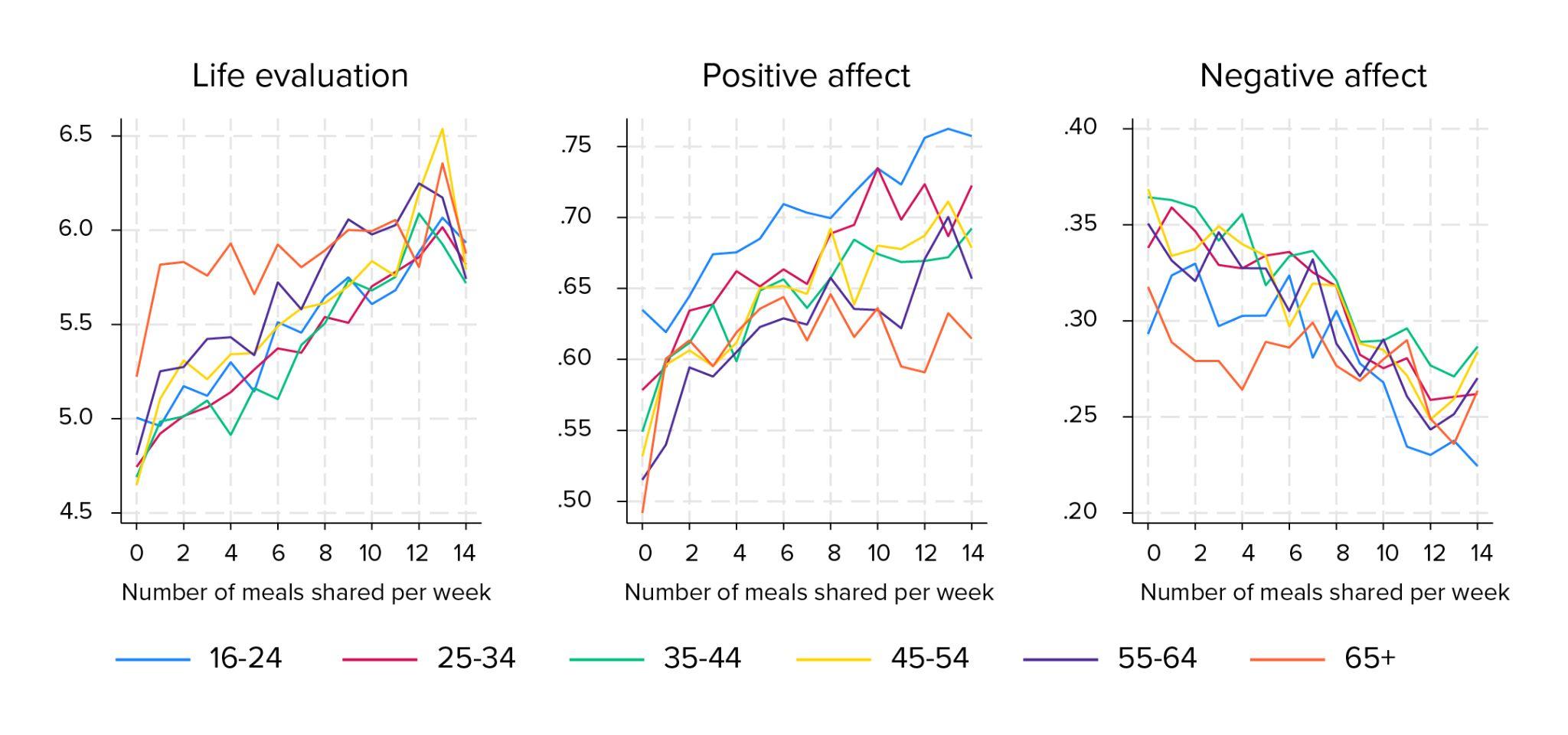


**Figure A9: Scatterplot of dining alone penalty and average number of shared meals (simple differences, by world region)**


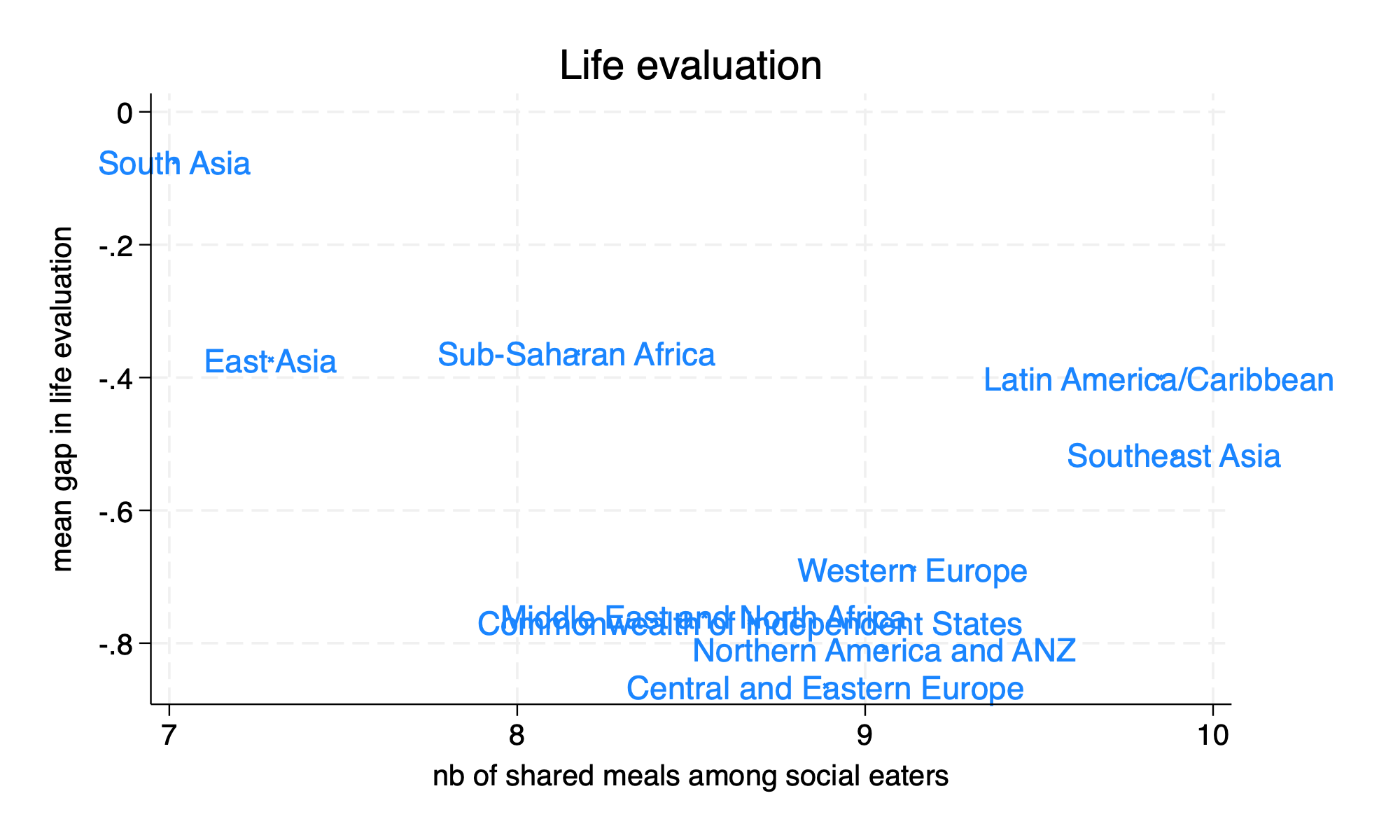


***Note:*** *Scatterplot, where each point is calculated from a world region. The x-axis reports the average number of shared meals per week among those who shared at least one meal. The y-axis reports the simple average difference in the life evaluation between those who dine alone and those who don't. Life evaluation is expressed on a 0-10 scale. Data includes the 2023 sample. Averages are calculated using country-level survey weights.*

**Figure A10: Scatterplot of dining alone penalty and average number of shared meals (regression coefficients, by country)**


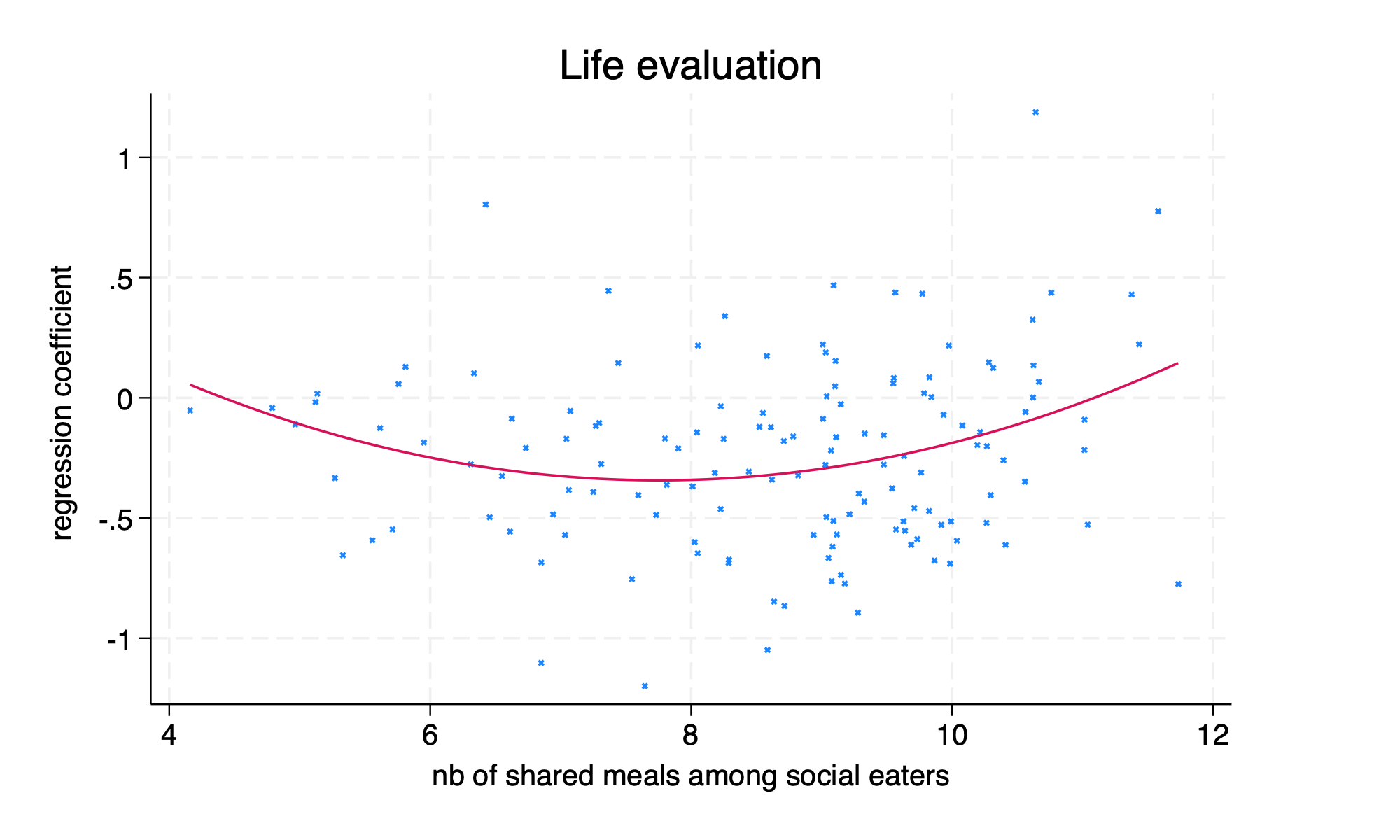


***Note:*** *Scatterplot with quadratic fit, where each point is calculated from a country. The x-axis reports the average number of shared meals per week among those who shared at least one meal. The y-axis reports the coefficients associated with the binary variable "dining alone” in 144 linear regressions where life evaluation is the dependent variable. Controls: income quintile, household size, gender, age, age-squared, education group, employment group, people’s ability to meet basic needs for food. Life evaluation is expressed on a 0-10 scale. Data includes the 2023 sample. Regressions use country-level survey weights.*

**Figure A11: Relationships between sharing meals, life evaluation, positive affect, and negative affect around the world by household size**

Gallup World Poll (2022-2023)


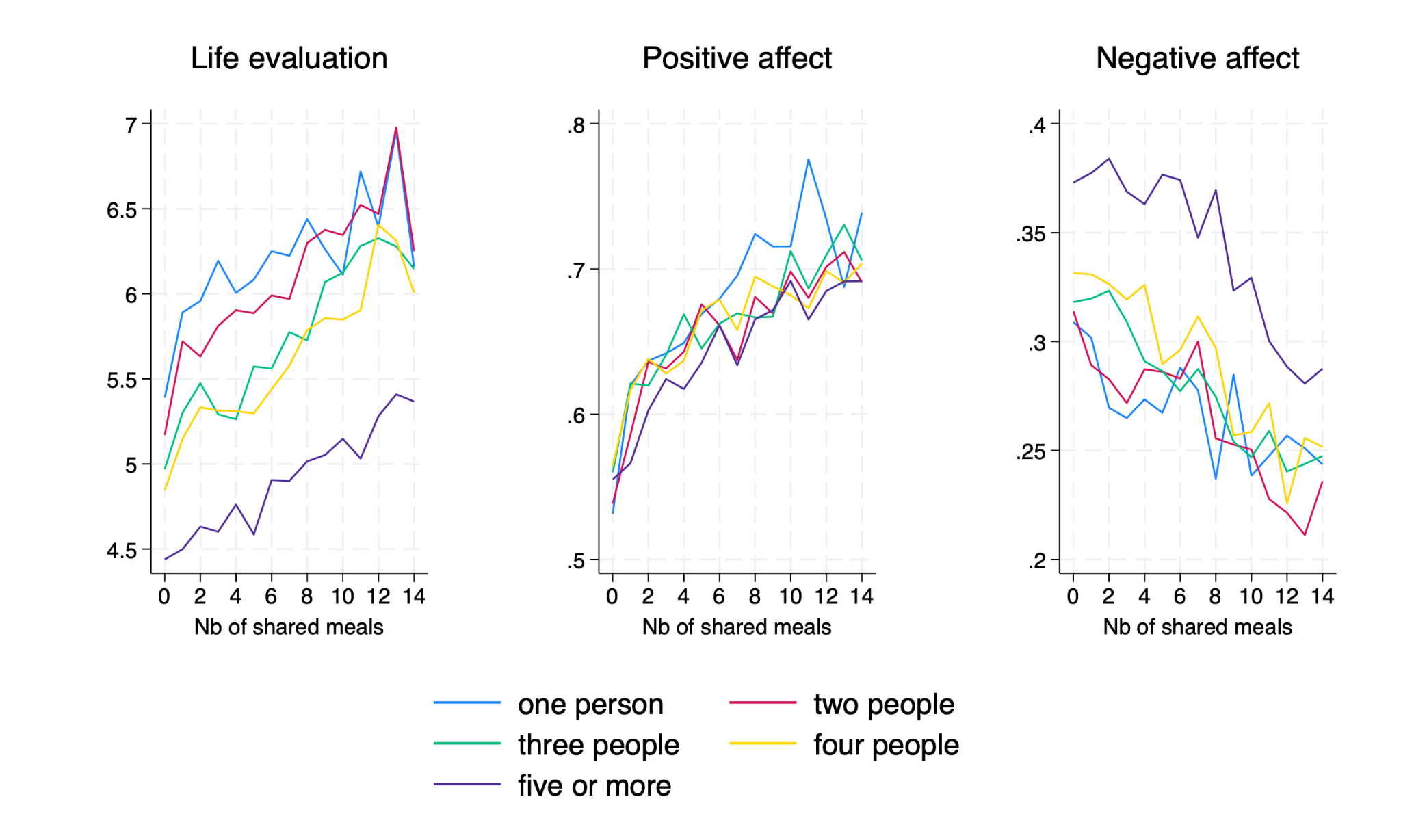


**Comment on Fig.A12-A15**

Lower life evaluations are predicted for those with relatively reduced opportunity of social connections - that is, those living alone (-0.381, p<0.001), not married/in a couple (-0.266, p<0.001), or unemployed ( -0.553, p<0.001). However, these social groups are also the ones who benefit the most from sharing extra meals. This is indicated by the relatively steeper line among those living alone (0.013, p=0.001), not married/in a couple (0.009, p<0.001), or unemployed (0.019, p=0.002). That is, the wellbeing gap between those with low and high opportunity of connections is reduced when sharing more meals.

It's important to notice that these global statistics mask stark differences across world regions. Figures A13-A15 show the same type of graph as Fig. A12, but decomposed by world region. To illustrate these differences, let us compare two regions which sometimes display diverging patterns: Western Europe and Latin America/Caribbean.

In Western Europe, the more meals are shared the smaller the wellbeing gap between those who are not in a couple (married or partnership) and those who are, while this is not observed in Latin America/Caribbean, where multi-generational households are more common. In Latin America/Caribbean the wellbeing gap of unemployment is greatly reduced by sharing extra meals. Instead, no such convergence is observed in Western Europe. Finally, both in Western Europe - where single-households are relatively common - and in Latin America/Caribbean - where single-households are rare - those living alone and those living with others seem to benefit equally from sharing additional meals.

**Figure A12: Predicted life evaluation among different social groups, by the number of shared meals (world)**

**
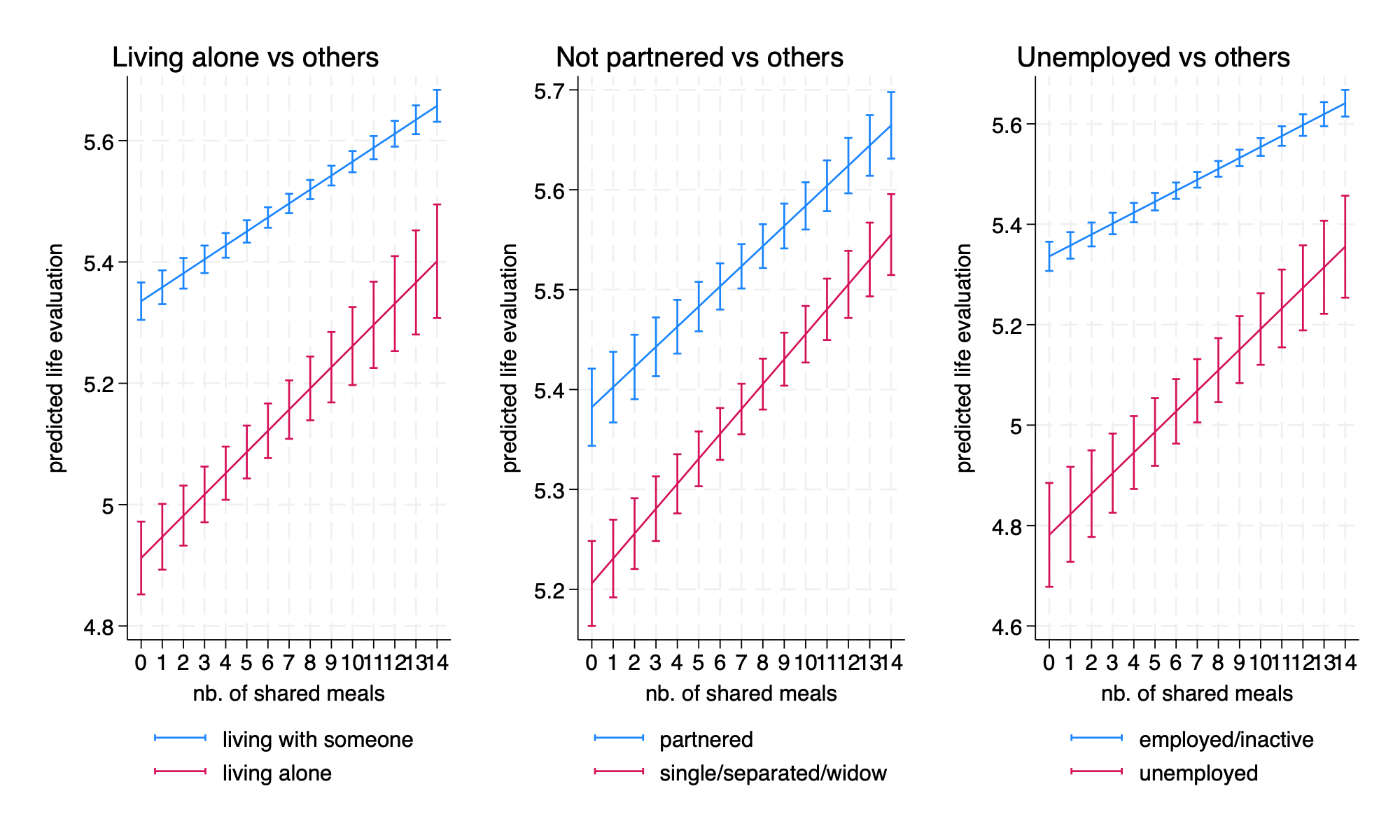
**

***Note:*** *Two separate regressions per image. Controls: country fixed effects, income quintile, household size (except in Panel A), gender, age, age-squared, education group, employment group (except in Panel C), people’s ability to meet basic needs for food. Life evaluation is expressed on a 0-10 scale. Data includes the 2023 sample. Predictive effect of the number of shared meals is assumed to be linear. Regressions use country-level survey weights.*

**Figure A13: Predicted life evaluation among those living alone vs others, by the number of shared meals (world regions)**

**
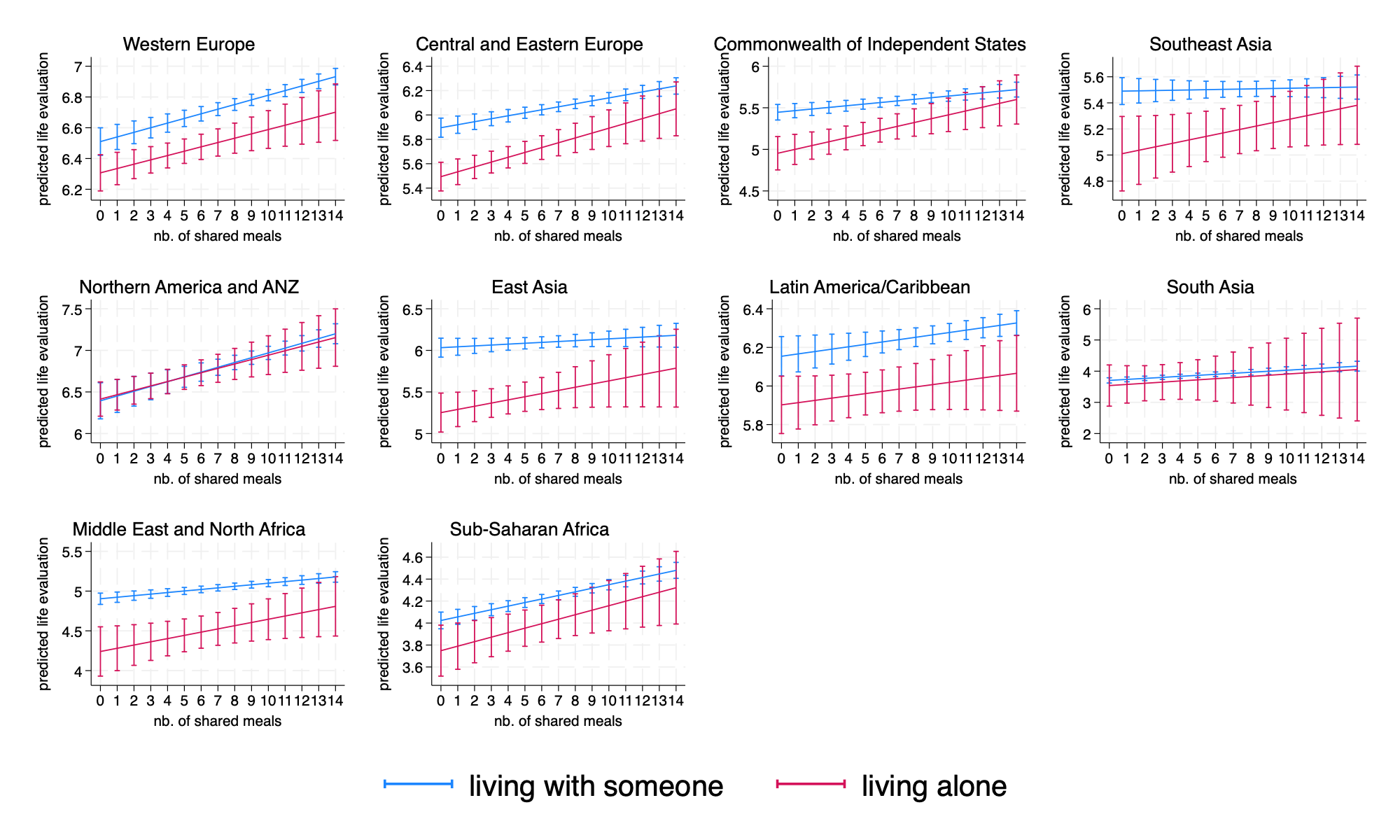
**

***Note:*** *Two separate regressions per image. Controls: country fixed effects, income quintile, gender, age, age-squared, education group, employment group, people’s ability to meet basic needs for food. Life evaluation is expressed on a 0-10 scale. Data includes the 2023 sample. Predictive effect of the number of shared meals is assumed to be linear. Regressions use country-level survey weights.*

**Figure A14: Predicted life evaluation among those not partnered vs others, by the number of shared meals (world regions)**

**
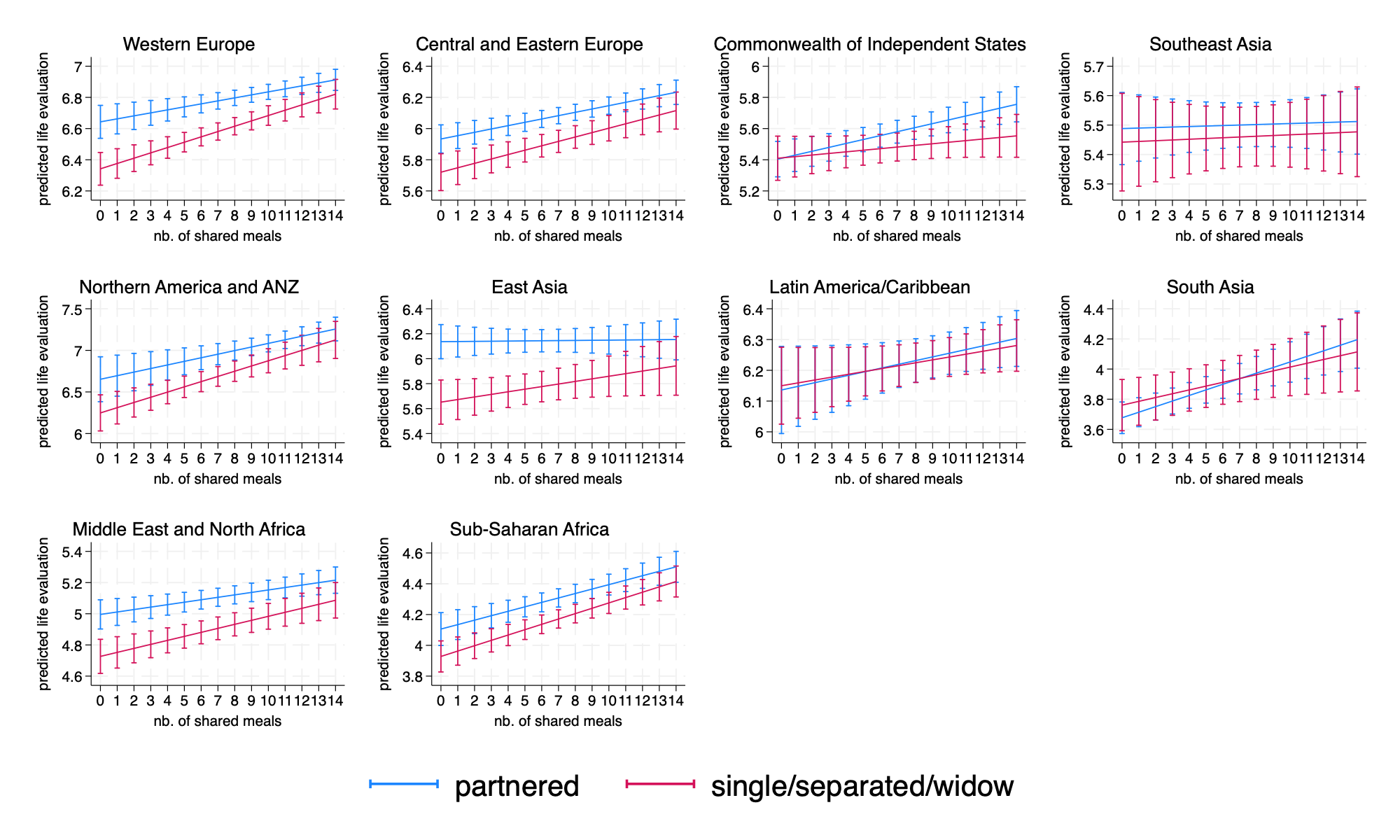
**

***Note:*** *Two separate regressions per image. Controls: country fixed effects, income quintile, household size, gender, age, age-squared, education group, employment group, people’s ability to meet basic needs for food. Life evaluation is expressed on a 0-10 scale. Data includes the 2023 sample. Predictive effect of the number of shared meals is assumed to be linear. Regressions use country-level survey weights.*

**Figure A15: Predicted life evaluation among those unemployed vs others, by the number of shared meals (world regions)**

**
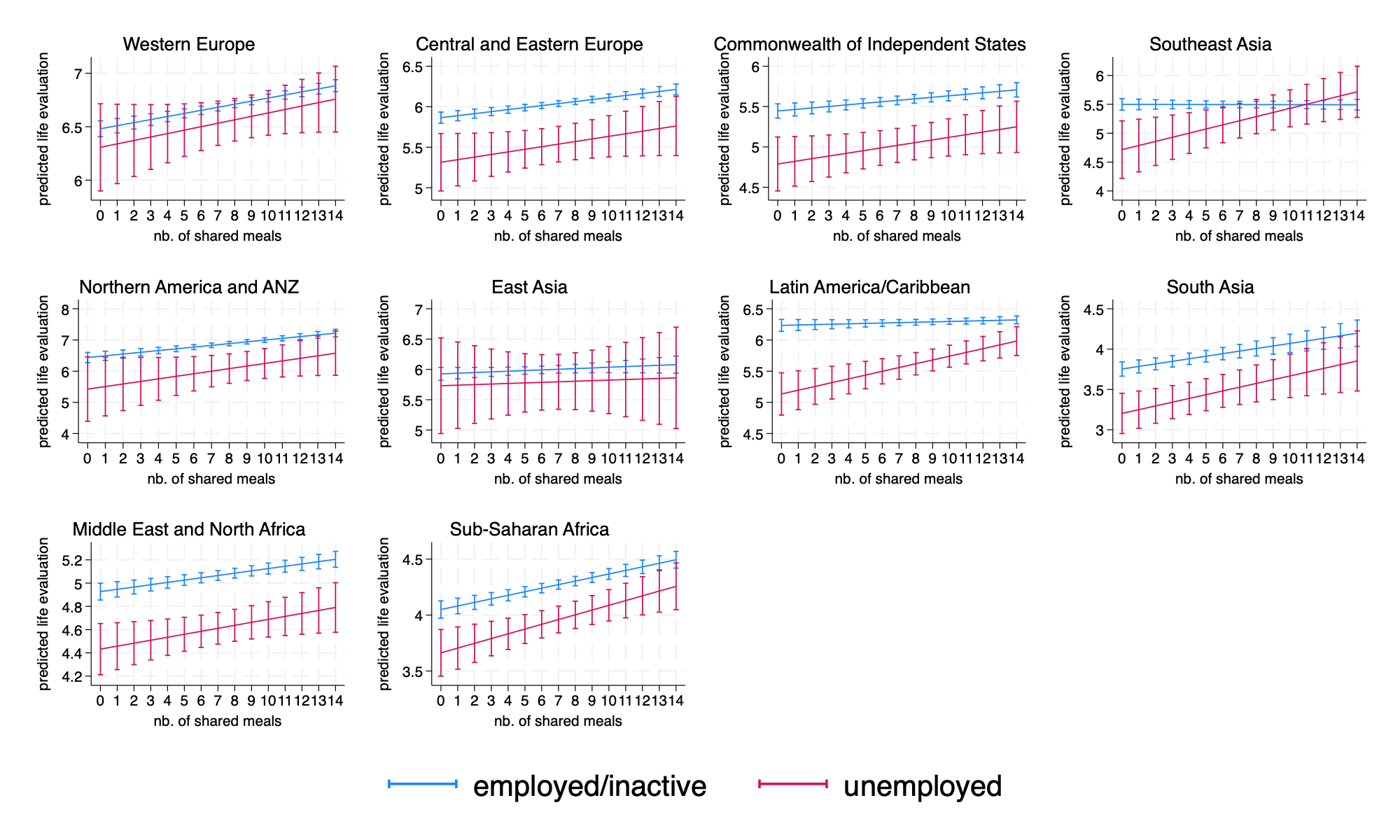
**

***Note:*** *Two separate regressions per image. Controls: country fixed effects, income quintile, household size, gender, age, age-squared, education group, people’s ability to meet basic needs for food. Life evaluation is expressed on a 0-10 scale. Data includes the 2023 sample. Predictive effect of the number of shared meals is assumed to be linear. Regressions use country-level survey weights.*

**Study 1 - Tables**

**Table A1:** Number of meals shared with others in the last week by region, gender and age

Gallup World Poll (2022-23)

| Category | **n** | **Mean** | **CI: LL** | **CI: UL** |
| --- | --- | --- | --- | --- |
| **Region** | | | | |
| Western Europe | 21535 | 8.3 | 8.2 | 8.4 |
| Central and Eastern Europe | 18511 | 7.3 | 7.2 | 7.5 |
| Commonwealth of Independent States | 11555 | 6.7 | 6.5 | 6.9 |
| Southeast Asia | 10009 | 7.7 | 7.5 | 7.9 |
| South Asia | 8012 | 3.9 | 3.7 | 4.1 |
| East Asia | 5491 | 5.6 | 5.5 | 5.8 |
| Latin America and Caribbean | 20036 | 8.8 | 8.7 | 8.9 |
| North America, Australia, and New Zealand | 4010 | 8.3 | 8.1 | 8.5 |
| Middle East and North Africa | 16972 | 7.1 | 6.9 | 7.2 |
| Sub-Saharan Africa | 36006 | 7.1 | 7.0 | 7.2 |
| **Gender** | | | | |
| Male | 70122 | 7.4 | 7.3 | 7.4 |
| Female | 82015 | 7.3 | 7.2 | 7.3 |
| **Age** | | | | |
| Under 30 | 44129 | 7.7 | 7.6 | 7.8 |
| 30-44 | 43342 | 7.4 | 7.4 | 7.5 |
| 45-59 | 31325 | 7.2 | 7.1 | 7.3 |
| 60+ | 33088 | 6.6 | 6.5 | 6.7 |
|  | | | | |

**Table A2:** Number of shared meals with others in the last week by country

Gallup World Poll (2022-23)

|  | **Country** | **Total meals shared per week** | **Dinners shared per week** | **Lunches shared per week** |
| --- | --- | --- | --- | --- |
| 1 | Senegal | 11.7 | 5.7 | 6.0 |
| 2 | Gambia | 11.2 | 5.2 | 6.0 |
| 3 | Malaysia | 11.0 | 5.6 | 5.4 |
| 4 | Paraguay | 10.8 | 5.2 | 5.6 |
| 5 | Poland | 10.7 | 5.1 | 5.6 |
| 6 | Iran | 10.6 | 5.2 | 5.4 |
| 7 | Ecuador | 10.4 | 5.2 | 5.2 |
| 8 | Taiwan | 10.2 | 5.5 | 4.7 |
| 9 | Iceland | 10.1 | 5.6 | 4.4 |
| 10 | Colombia | 10.1 | 5.1 | 5.0 |
| 11 | Kazakhstan | 10.0 | 5.1 | 4.9 |
| 12 | Uruguay | 9.8 | 4.9 | 4.9 |
| 13 | Thailand | 9.8 | 5.3 | 4.6 |
| 14 | Peru | 9.7 | 4.8 | 4.9 |
| 15 | Mexico | 9.7 | 4.8 | 4.9 |
| 16 | Morocco | 9.6 | 4.5 | 5.0 |
| 17 | Slovakia | 9.5 | 4.7 | 4.7 |
| 18 | Portugal | 9.5 | 4.9 | 4.7 |
| 19 | Sweden | 9.5 | 5.1 | 4.4 |
| 20 | Mali | 9.5 | 4.6 | 4.9 |
| 21 | Yemen | 9.5 | 4.5 | 4.9 |
| 22 | Brazil | 9.4 | 4.6 | 4.8 |
| 23 | Argentina | 9.3 | 4.7 | 4.7 |
| 24 | Cyprus | 9.2 | 4.6 | 4.5 |
| 25 | South Africa | 9.2 | 5.1 | 4.1 |
| 26 | Costa Rica | 9.2 | 4.6 | 4.6 |
| 27 | Venezuela | 9.2 | 4.5 | 4.6 |
| 28 | Serbia | 9.1 | 4.3 | 4.7 |
| 29 | Spain | 9.0 | 4.7 | 4.4 |
| 30 | Indonesia | 9.0 | 4.4 | 4.5 |
| 31 | Italy | 8.9 | 4.7 | 4.3 |
| 32 | Malta | 8.9 | 5.0 | 3.9 |
| 33 | Denmark | 8.9 | 4.9 | 4.0 |
| 34 | Netherlands | 8.8 | 4.8 | 4.0 |
| 35 | Uganda | 8.8 | 4.4 | 4.5 |
| 36 | Bolivia | 8.8 | 4.0 | 4.8 |
| 37 | Norway | 8.8 | 4.9 | 3.9 |
| 38 | Slovenia | 8.7 | 3.8 | 4.9 |
| 39 | Czech Republic | 8.7 | 4.4 | 4.3 |
| 40 | Singapore | 8.7 | 4.5 | 4.2 |
| 41 | Dominican Republic | 8.7 | 4.3 | 4.3 |
| 42 | Uzbekistan | 8.7 | 4.3 | 4.4 |
| 43 | Tajikistan | 8.7 | 4.3 | 4.4 |
| 44 | Ireland | 8.6 | 4.7 | 4.0 |
| 45 | Bosnia Herzegovina | 8.6 | 4.1 | 4.5 |
| 46 | Luxembourg | 8.5 | 4.4 | 4.2 |
| 47 | Moldova | 8.5 | 4.6 | 3.9 |
| 48 | Australia | 8.5 | 4.9 | 3.6 |
| 49 | Mozambique | 8.5 | 4.1 | 4.3 |
| 50 | Israel | 8.4 | 4.4 | 4.0 |
| 51 | Hungary | 8.4 | 4.2 | 4.2 |
| 52 | New Zealand | 8.4 | 4.9 | 3.5 |
| 53 | Eswatini | 8.4 | 4.5 | 3.9 |
| 54 | Canada | 8.4 | 4.8 | 3.6 |
| 55 | Laos | 8.4 | 4.4 | 4.0 |
| 56 | Zambia | 8.4 | 4.3 | 4.1 |
| 57 | Montenegro | 8.4 | 3.8 | 4.6 |
| 58 | Palestine | 8.4 | 3.4 | 5.0 |
| 59 | Chile | 8.4 | 3.4 | 5.0 |
| 60 | Panama | 8.3 | 4.3 | 4.1 |
| 61 | Mauritania | 8.3 | 4.1 | 4.2 |
| 62 | Belgium | 8.3 | 4.4 | 4.0 |
| 63 | Tanzania | 8.3 | 4.3 | 4.0 |
| 64 | Mauritius | 8.3 | 5.1 | 3.2 |
| 65 | Romania | 8.2 | 4.2 | 4.0 |
| 66 | Kenya | 8.1 | 4.7 | 3.4 |
| 67 | Austria | 8.1 | 4.0 | 4.0 |
| 68 | Switzerland | 8.1 | 4.2 | 3.9 |
| 69 | Hong Kong | 8.0 | 4.4 | 3.6 |
| 70 | United States | 7.9 | 4.7 | 3.2 |
| 71 | El Salvador | 7.8 | 4.0 | 3.8 |
| 72 | Finland | 7.7 | 3.8 | 3.9 |
| 73 | Armenia | 7.6 | 3.7 | 3.9 |
| 74 | Guatemala | 7.6 | 3.8 | 3.8 |
| 75 | United Arab Emirates | 7.6 | 3.8 | 3.8 |
| 76 | Kosovo | 7.6 | 3.5 | 4.0 |
| 77 | Croatia | 7.5 | 3.2 | 4.3 |
| 78 | Greece | 7.5 | 3.2 | 4.3 |
| 79 | Egypt | 7.5 | 3.6 | 3.9 |
| 80 | Kuwait | 7.5 | 3.6 | 3.9 |
| 81 | Namibia | 7.5 | 4.1 | 3.4 |
| 82 | United Kingdom | 7.5 | 4.2 | 3.3 |
| 83 | Guinea | 7.4 | 3.9 | 3.4 |
| 84 | Latvia | 7.3 | 3.8 | 3.5 |
| 85 | Myanmar | 7.2 | 3.5 | 3.6 |
| 86 | Madagascar | 7.1 | 3.6 | 3.5 |
| 87 | Russia | 7.1 | 3.6 | 3.5 |
| 88 | Chad | 7.1 | 3.8 | 3.3 |
| 89 | Honduras | 7.1 | 3.5 | 3.6 |
| 90 | France | 7.0 | 3.6 | 3.4 |
| 91 | Germany | 7.0 | 3.6 | 3.4 |
| 92 | North Macedonia | 7.0 | 3.0 | 4.0 |
| 93 | Nigeria | 6.9 | 4.1 | 2.8 |
| 94 | Afghanistan | 6.9 | 3.5 | 3.4 |
| 95 | Niger | 6.9 | 3.6 | 3.4 |
| 96 | Zimbabwe | 6.8 | 4.4 | 2.4 |
| 97 | Tunisia | 6.8 | 3.4 | 3.4 |
| 98 | Togo | 6.5 | 3.7 | 2.8 |
| 99 | Turkey | 6.5 | 3.8 | 2.6 |
| 100 | Algeria | 6.3 | 3.1 | 3.2 |
| 101 | Jamaica | 6.3 | 4.3 | 2.0 |
| 102 | Ivory Coast | 6.3 | 3.6 | 2.7 |
| 103 | Nicaragua | 6.2 | 3.1 | 3.2 |
| 104 | Gabon | 6.2 | 3.4 | 2.8 |
| 105 | Botswana | 6.2 | 3.5 | 2.7 |
| 106 | Vietnam | 6.2 | 3.0 | 3.2 |
| 107 | Lesotho | 6.0 | 3.2 | 2.8 |
| 108 | Saudi Arabia | 5.9 | 2.9 | 3.1 |
| 109 | Cameroon | 5.8 | 3.0 | 2.8 |
| 110 | Iraq | 5.7 | 2.8 | 2.9 |
| 111 | Comoros | 5.6 | 3.2 | 2.4 |
| 112 | Kyrgyzstan | 5.5 | 2.6 | 2.9 |
| 113 | Ethiopia | 5.5 | 2.6 | 2.9 |
| 114 | Sierra Leone | 5.4 | 3.2 | 2.2 |
| 115 | Moldova | 5.3 | 2.6 | 2.6 |
| 116 | Malawi | 5.2 | 2.5 | 2.7 |
| 117 | Lebanon | 5.1 | 2.0 | 3.1 |
| 118 | Ghana | 5.0 | 3.1 | 1.9 |
| 119 | Cambodia | 4.9 | 2.4 | 2.5 |
| 120 | Albania | 4.7 | 2.2 | 2.4 |
| 121 | Congo Kinshasa | 4.5 | 3.0 | 1.5 |
| 122 | Azerbaijan | 4.5 | 2.3 | 2.2 |
| 123 | Bulgaria | 4.4 | 2.2 | 2.2 |
| 124 | Congo Brazzaville | 4.3 | 2.3 | 2.0 |
| 125 | South Korea | 4.3 | 1.6 | 2.7 |
| 126 | Liberia | 4.3 | 2.3 | 2.0 |
| 127 | Libya | 4.1 | 2.1 | 2.1 |
| 128 | Ukraine | 4.1 | 1.7 | 2.4 |
| 129 | Philippines | 4.0 | 1.8 | 2.3 |
| 130 | Benin | 4.0 | 2.3 | 1.8 |
| 131 | India | 4.0 | 2.0 | 2.0 |
| 132 | Japan | 3.7 | 1.8 | 1.9 |
| 133 | Jordan | 3.4 | 1.4 | 2.0 |
| 134 | Georgia | 3.3 | 1.9 | 1.5 |
| 135 | Nepal | 3.3 | 1.7 | 1.6 |
| 136 | Sri Lanka | 3.2 | 1.4 | 1.8 |
| 137 | Pakistan | 3.1 | 1.4 | 1.7 |
| 138 | Mongolia | 3.0 | 1.1 | 1.9 |
| 139 | Lithuania | 2.9 | 1.3 | 1.6 |
| 140 | Estonia | 2.7 | 1.1 | 1.6 |
| 141 | Bangladesh | 2.7 | 1.3 | 1.4 |

| **Table A3:** Dining alone and life evaluations around the world  Gallup World Poll (2022,2023) | | | | | | | | | |  |
| --- | --- | --- | --- | --- | --- | --- | --- | --- | --- | --- |
|  | Western Europe | Central and Eastern Europe | Commonwealth of Independent States | Southeast Asia | Northern America and ANZ | East Asia | Latin America/Caribbean | South Asia | Middle East and North Africa | Sub-Saharan Africa |
|  | (1) | (2) | (3) | (4) | (5) | (6) | (7) | (8) | (9) |  |
|  | Life evaluation (0-10) | | | | | | | | |  |
| **Dining alone** | -0.393*** | -0.264*** | -0.351*** | -0.145* | -0.772*** | -0.193** | -0.177** | -0.312*** | -0.312*** | -0.155** |
|  | (0.067) | (0.053) | (0.067) | (0.075) | (0.145) | (0.086) | (0.072) | (0.074) | (0.054) | (0.062) |
| **Observations** | 21,219 | 18,704 | 10,049 | 9,528 | 3,874 | 5,417 | 18,610 | 7,676 | 16,504 | 33,394 |
| **R-squared** | 0.184 | 0.240 | 0.140 | 0.168 | 0.174 | 0.149 | 0.102 | 0.247 | 0.327 | 0.105 |
| Life evaluations measured using the Cantril Ladder on a scale from 0 to 10. Individual-level data from the Gallup World Poll. Dining alone indicates eating all meals alone the previous week. Controlling for country fixed effects, income quintile, household size, gender, age, age-squared, education group, employment group, people’s ability to meet basic needs for food. Standard errors in parentheses. Data weighted to be nationally representative. *** p<.01, ** p<.05, * p<.1 | | | | | | | | | |  |

**Table A4:** Household size, sharing meals and life evaluations around the world

Gallup World Poll (2022,2023)

----------------------------------------------
 Life evaluation (0-10)
----------------------------------------------
nb. of shared meals 0.036***
 (0.005)
**HH1** ref. category
 (.)
**HH2** 0.315***
 (0.041)
**HH3** 0.453***
 (0.045)
**HH4** 0.602***
 (0.046)
**HH5+** 0.611***
 (0.044)
**HH1 # nb. of shared meals** ref. category

(.)

**HH2 # nb. of shared meals** -0.016***
 (0.005)
**HH3 # nb. of shared meals** -0.011**
 (0.006)
**HH4 # nb. of shared meals** -0.018***
 (0.006)
**HH5+ # nb. of shared meals** -0.013**
 (0.005)

**Observations** 144,975

**R squared**  0.271

----------------------------------------------

Life evaluations measured using the Cantril Ladder on a scale from 0 to 10. Individual-level data from the Gallup World Poll. HH1, HH2, HH3, HH4, HH5+ refer to the respective sizes of the household. Controlling for country fixed effects, income quintile, household size, gender, age, age-squared, education group, employment group, people’s ability to meet basic needs for food. Standard errors in parentheses. Data weighted to be nationally representative. *** p<.01, ** p<.05, * p<.1.

# **Study 2 - Figures**

**Figure B1:** Robustness checks using alternative measures of meal sharing

American TIme Use Survey (ATUS)

*Panel A:* Percent dining alone including time spent on food preparation


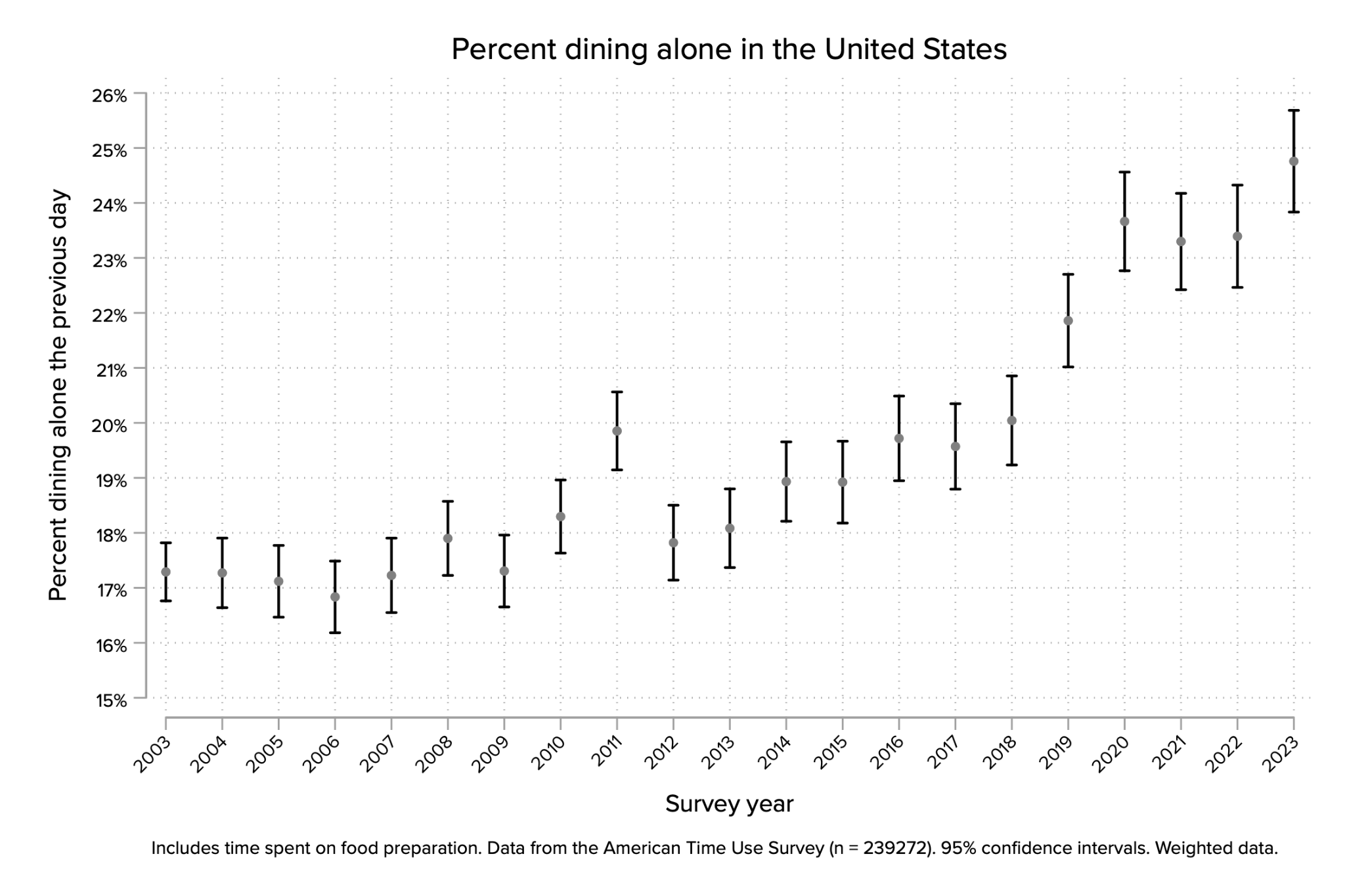


*Panel B:* Percent of time alone while dining


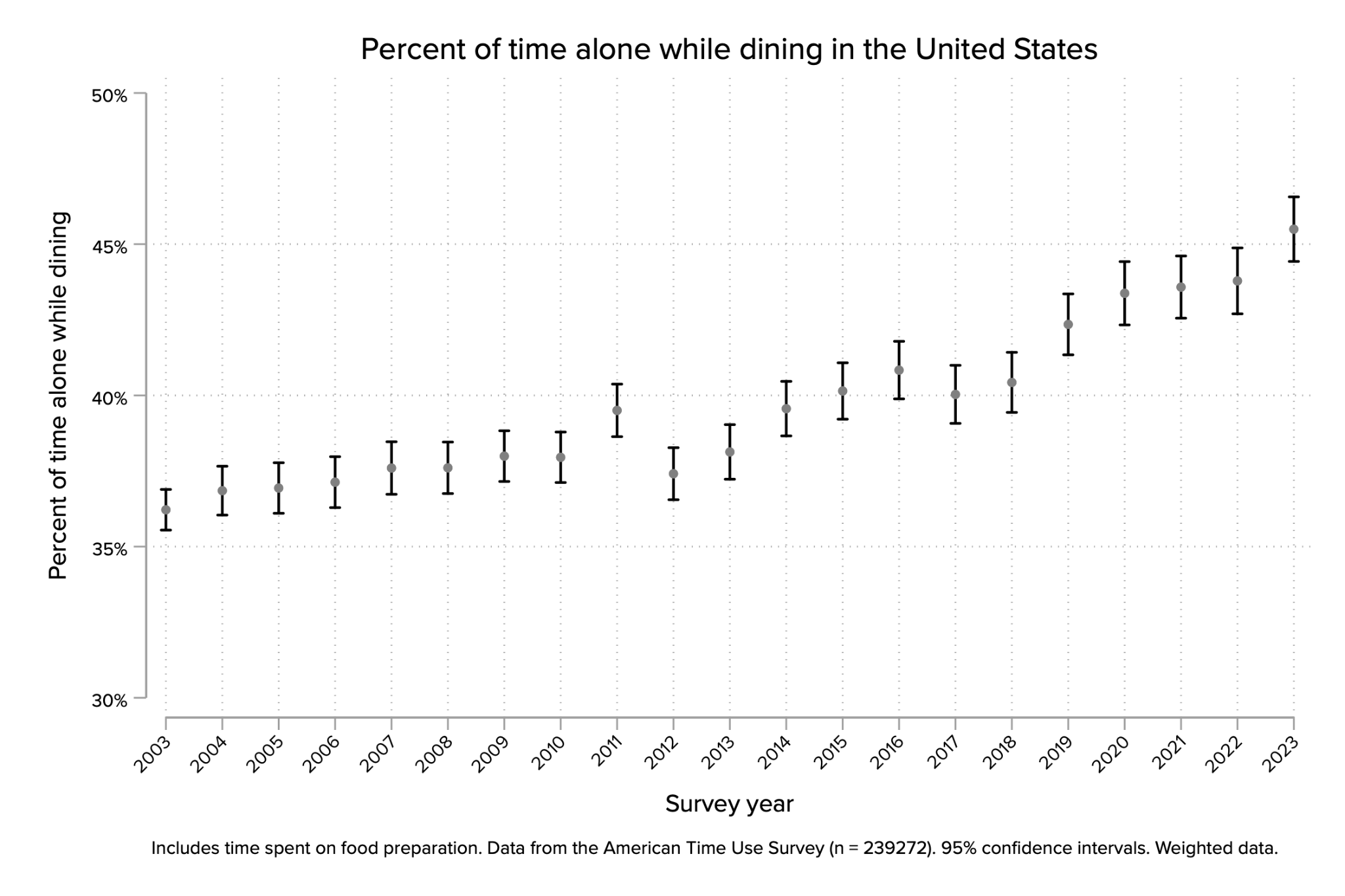


*Panel C:* Percent of time alone while dining including time spent on food preparation


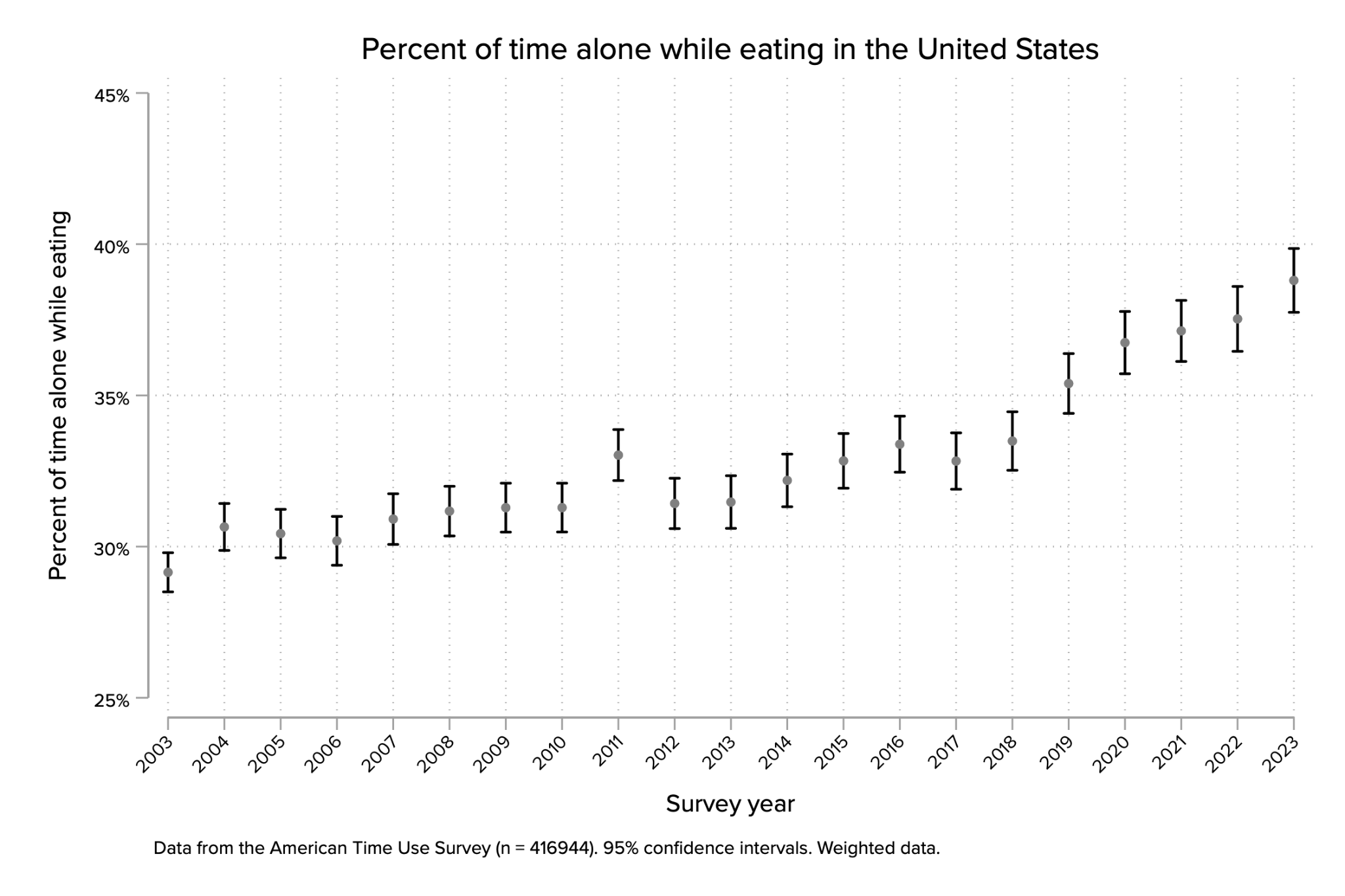


**Note:** Data from the American Time Use Survey weighted to be representative of the general population. 95% confidence intervals.

**Figure B2:** Percent living alone in the United States

American Time Use Survey (ATUS)


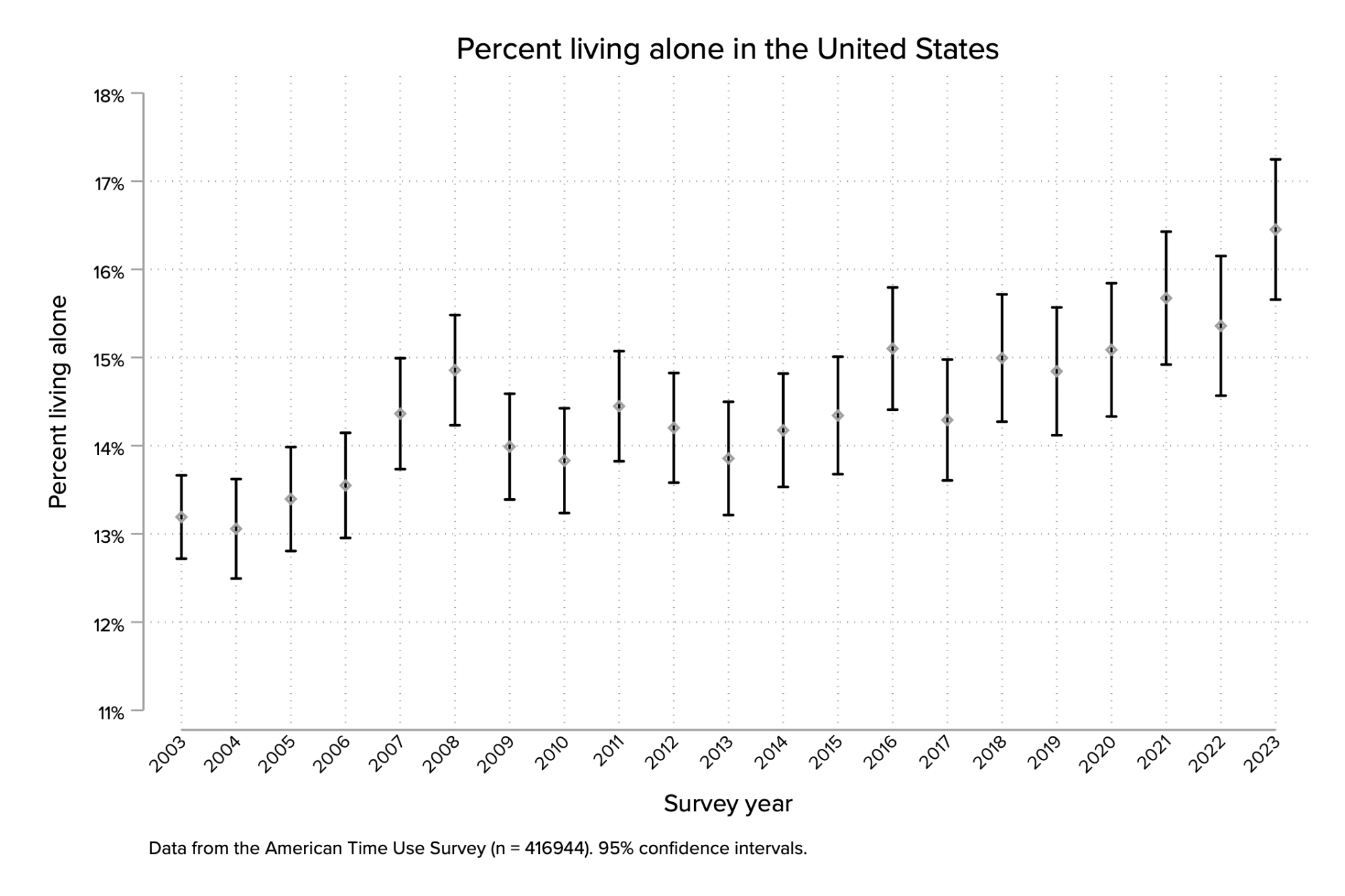


**Figure B3:** Estimating changing in dining alone in the USA controlling for other factors

American Time Use Survey (ATUS)


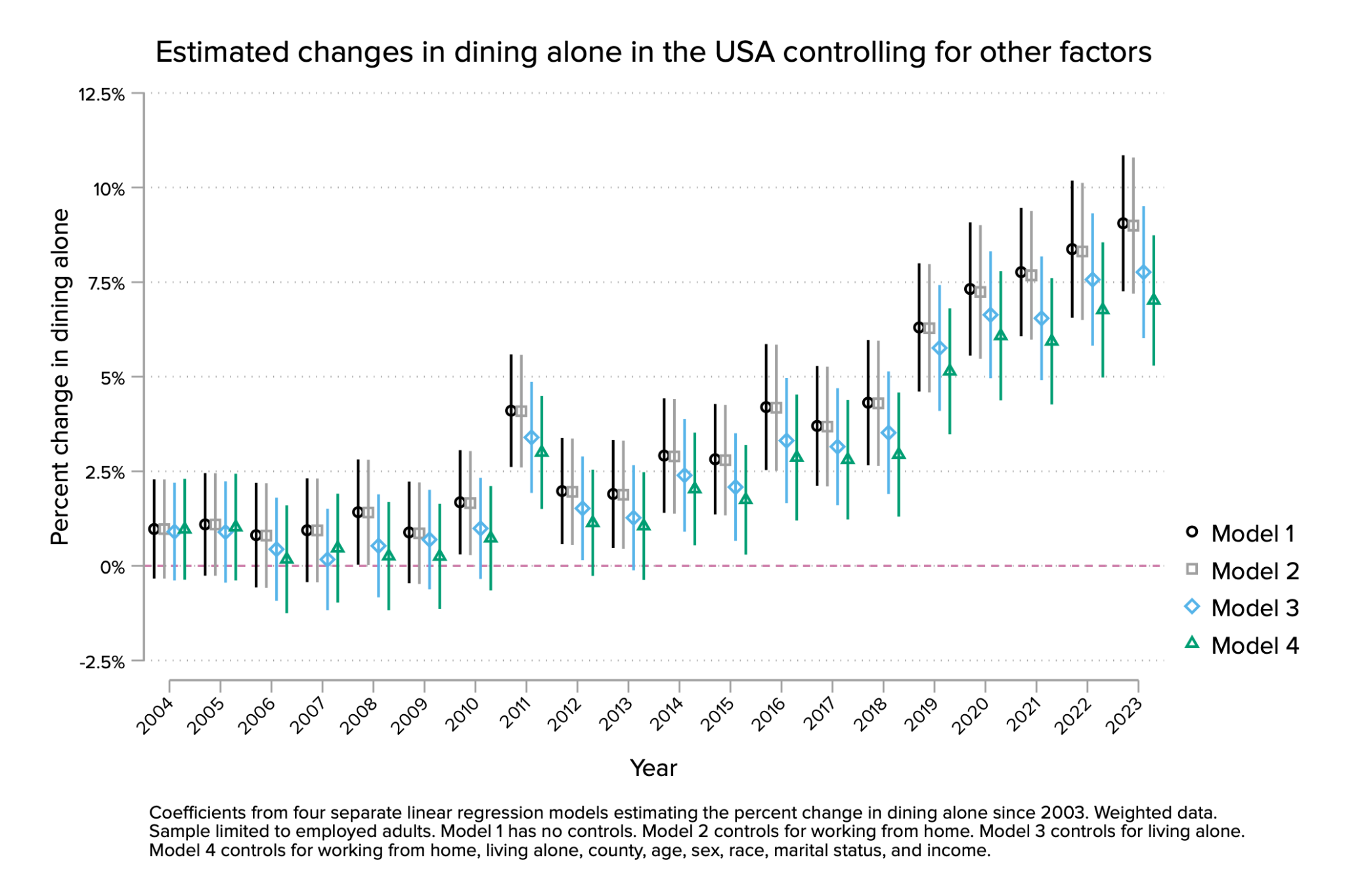


**Note:** Coefficients from four separate linear regression models estimating the percent change in dining alone since 2003. Data weighted to be representative of the general population. Sample limited to employed adults. Model 1 has no controls. Model 2 controls for working from home. Model 3 controls for living alone. Model 4 controls for working from home, living alone, county, age, sex, race, marital status, and income.

**Figure B4: Percent of employed adults working from home**
ATUS, 2003–2023


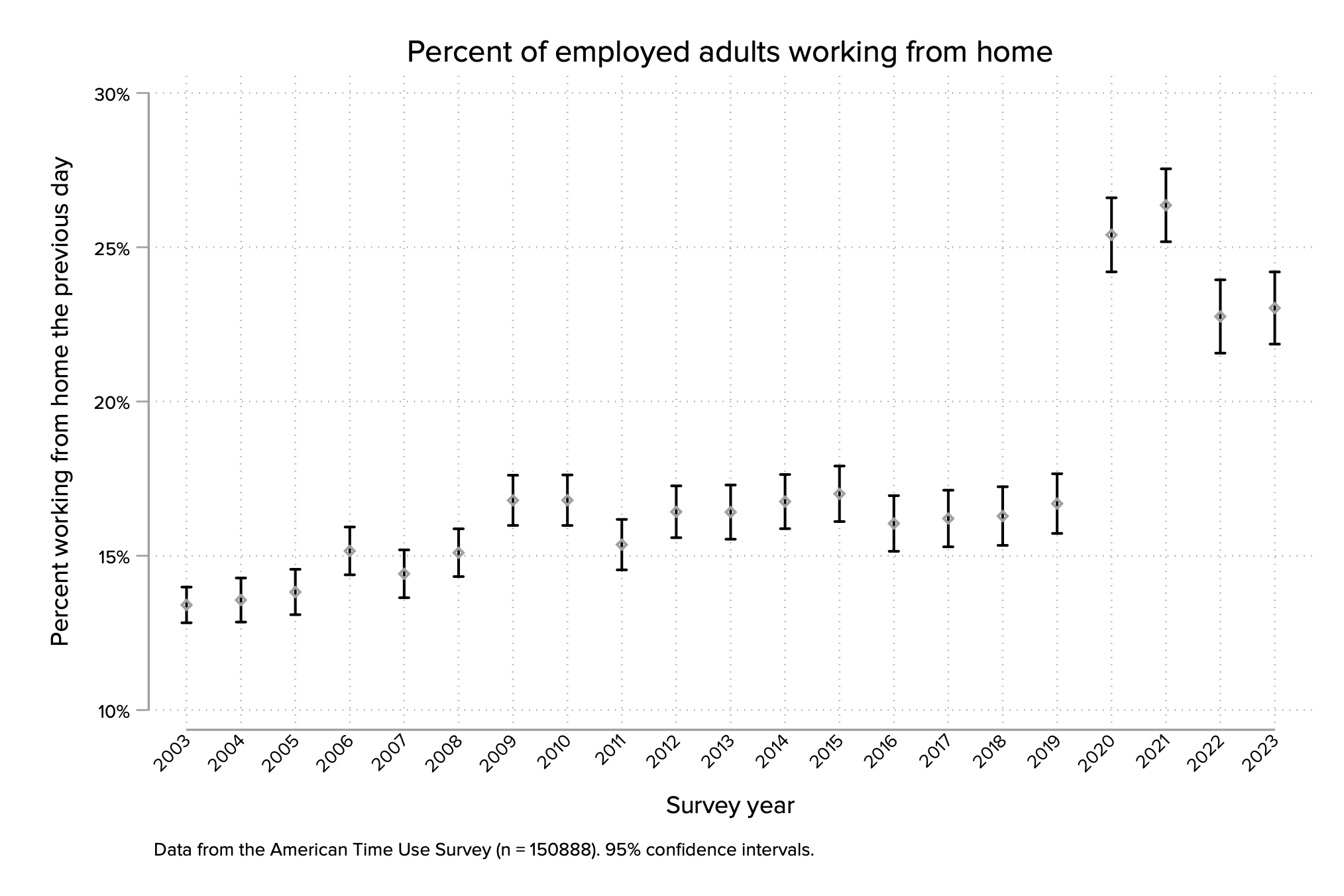


***Note:*** *Data from the American Time Use Survey weighted to be representative of the general population. Sample limited to employed adults (n = 150,888). 95% confidence intervals displayed. Remote work estimated as the percent of respondents who report spending at least some of their time working from home in the previous day..*

**Figure B5:  Dining alone in the United States, by remote work**
ATUS, 2003–2023


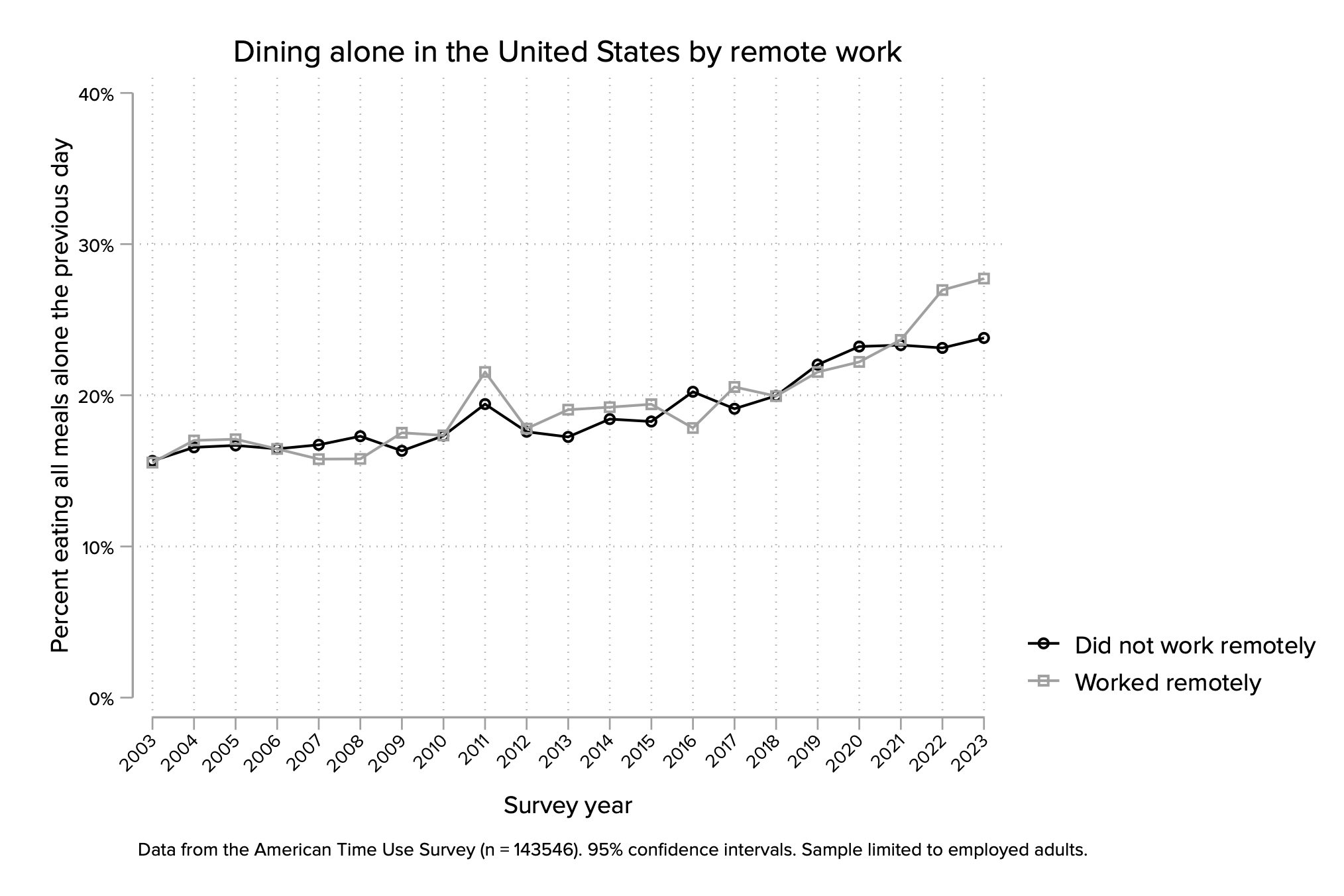


***Note:*** *Data from the American Time Use Survey weighted to be representative of the general population. Sample limited to employed adults (n = 143,546). Working remotely measured as the percent of employed adults who report working from home at least part of the time in the previous day. Dining alone measured as the share of respondents in each survey year reporting eating all meals alone in the previous day. 95% confidence intervals displayed.*

**Figure B6:  Dining alone in the United States by gender**
ATUS, 2003–2023


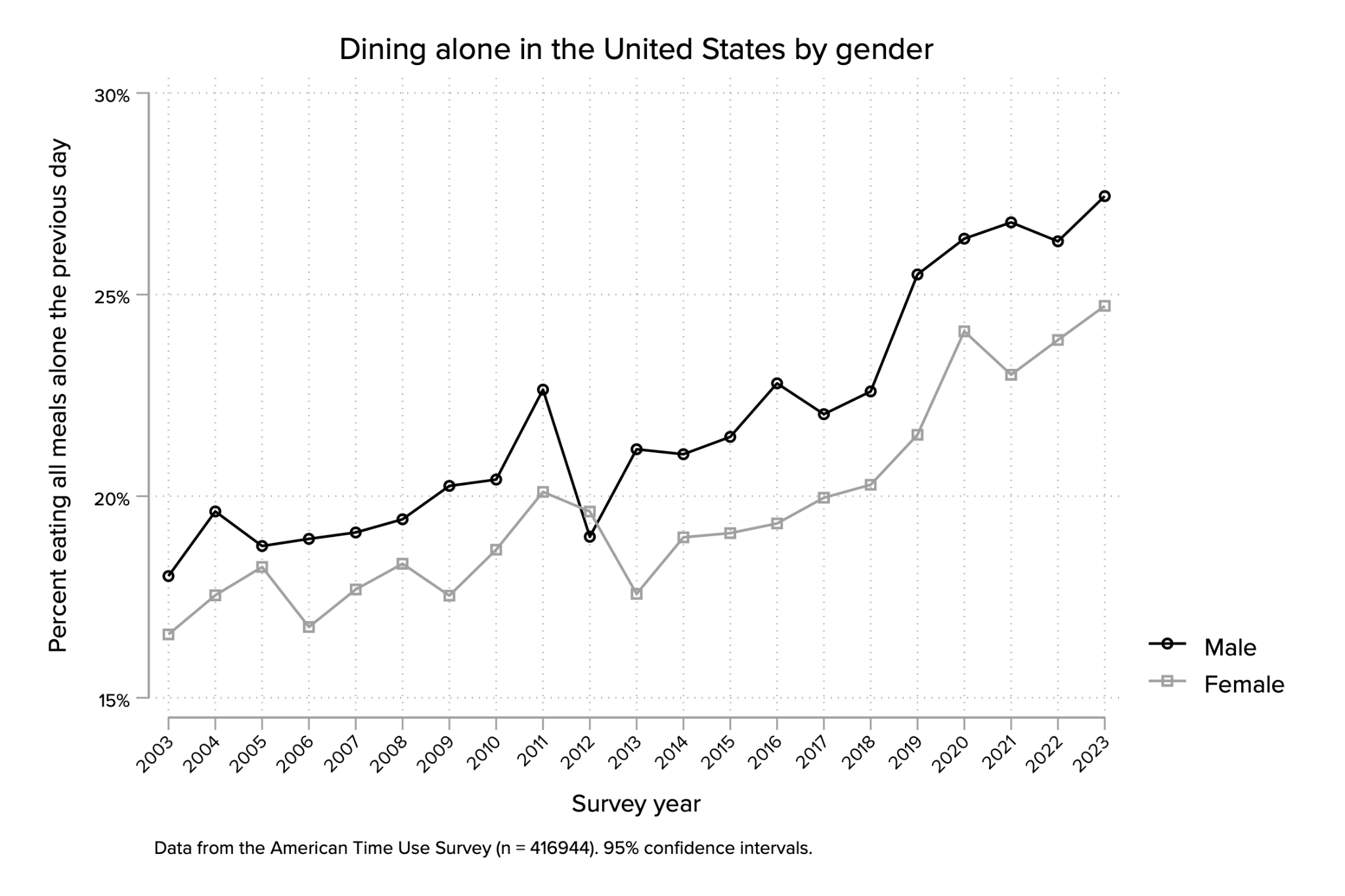


***Note:*** *Data from the American Time Use Survey weighted to be representative of the general population (n = 234,185). 95% confidence intervals displayed. Dining alone measured as the share of respondents in each survey year reporting eating all meals alone in the previous day.*

| **Study 2 - Tables** **Table B1:** Dining alone and life evaluations in the United States  American Time Use Survey (2012, 2013, 2021) | | | | | | | | | |
| --- | --- | --- | --- | --- | --- | --- | --- | --- | --- |
|  | Overall | Male | Female | 18-24 | 25-34 | 35-44 | 45-54 | 55-64 | 65+ |
|  | (1) | (2) | (3) | (4) | (5) | (6) | (7) | (8) | (9) |
|  | Life evaluation (0-10) | | | | | | | | |
| Dining alone | -.272*** | -.279*** | -.274*** | -.319** | -.222** | -.183* | -.264** | -.422*** | -.354*** |
|  | (.042) | (.062) | (.057) | (.14) | (.113) | (.104) | (.105) | (.098) | (.082) |
|  |  |  |  |  |  |  |  |  |  |
| Observations | 27556 | 12307 | 15210 | 2424 | 4222 | 5197 | 4595 | 4538 | 6253 |
| R-squared | .078 | .106 | .095 | .158 | .169 | .152 | .137 | .142 | .104 |
| Life evaluations measured using the Cantril Ladder on a scale from 0 to 10. Individual-level data from the American Time Use Survey (2012, 2013, 2021). Dining alone indicates eating all meals alone the previous day. Controlling for age, sex, county, marital status, and race. Standard errors in parentheses. Data weighted to be nationally representative. *** p<.01, ** p<.05, * p<.1 | | | | | | | | | |

| **Table B2:** Dining alone and affect in the United States  American Time Use Survey (2012, 2013, 2021) | | | | | |
| --- | --- | --- | --- | --- | --- |
|  | (1) | (2) | (3) | (4) | (5) |
|  | Happy | Pain | Sad | Tired | Stress |
| Dining alone | -.493*** | .124*** | .209*** | .001 | .134*** |
|  | (.041) | (.041) | (.036) | (.049) | (.045) |
|  |  |  |  |  |  |
| Observations | 18754 | 18809 | 18795 | 18810 | 18814 |
| R-squared | .069 | .101 | .067 | .066 | .063 |
| Affect variables measured on a scale from 1 to 6. Dining alone indicates eating all meals alone the previous day. Activity-level data from the American Time Use Survey (2010, 2012, 2013, 2021). Dining alone indicates eating all meals alone the previous day. Controlling for age, sex, county, marital status, and race. Standard errors in parentheses. Data weighted to be nationally representative. *** p<.01, ** p<.05, * p<.1 | | | | | |

| **Table B3:** Dining alone, life evaluation, and affect (standardized)  American Time Use Survey (2012, 2013, 2021) | | | | | | |
| --- | --- | --- | --- | --- | --- | --- |
|  | (1) | (2) | (3) | (4) | (5) | (6) |
|  | Life evaluation | Happy | Stress | Sad | Tired | Pain |
| Dining alone | -.136*** | -.255*** | .127*** | .157*** | .04** | .102*** |
|  | (.021) | (.018) | (.019) | (.018) | (.018) | (.017) |
|  |  |  |  |  |  |  |
| Observations | 27556 | 39810 | 39853 | 39848 | 39856 | 39854 |
| R-squared | .078 | .049 | .05 | .043 | .058 | .074 |
| All dependent variables averaged at the individual-level standardized with mean 0 and standard deviation 1. Individual-level data from the American Time Use Survey (2012, 2013, 2021). Dining alone indicates eating all meals alone the previous day. Controlling for age, sex, county, marital status, and race. Standard errors in parentheses. Data weighted to be nationally representative.*** p<.01, ** p<.05, * p<.1 | | | | | | |

**Table B4:** Dining alone and life evaluation across demographic groups

American Time Use Survey (2012, 2013, 2021)

|  | (1) | (2) | (3) | (4) | (5) |
| --- | --- | --- | --- | --- | --- |
|  | Life evaluation (0-10) | | | | |
| Dining alone | -.272*** | -.257*** | -.227*** | -.238*** | -.233*** |
|  | (.042) | (.052) | (.051) | (.055) | (.058) |
| Married |  | .634*** |  |  |  |
|  |  | (.041) |  |  |  |
| Married *x* dining alone |  | -.064 |  |  |  |
|  |  | (.087) |  |  |  |
| Unemployed |  |  | -.325*** |  |  |
|  |  |  | (.045) |  |  |
| Unemployed *x* dining alone |  |  | -.111 |  |  |
|  |  |  | (.08) |  |  |
| Lives alone |  |  |  | -.047 |  |
|  |  |  |  | (.066) |  |
| Lives alone *x* dining alone |  |  |  | -.067 |  |
|  |  |  |  | (.085) |  |
| Remote work |  |  |  |  | -.018 |
|  |  |  |  |  | (.049) |
| Remote work *x* dining alone |  |  |  |  | -.029 |
|  |  |  |  |  | (.113) |
|  |  |  |  |  |  |
| County | X | X | X | X | X |
| Age | X | X | X | X | X |
| Race | X | X | X | X | X |
| Gender | X | X | X | X | X |
| Marital status | X | - | X | X | X |
| Employment status | X | X | - | X | - |
|  |  |  |  |  |  |
| Constant | 7.246*** | 6.917*** | 7.374*** | 7.252*** | 7.256*** |
|  | (.018) | (.031) | (.023) | (.02) | (.023) |
| Observations | 27556 | 27556 | 27556 | 27556 | 16463 |
| R-squared | .083 | .082 | .083 | .083 | .095 |
| Life evaluations measured using the Cantril Ladder on a scale from 0 to 10. Individual-level data from the American Time Use Survey (2012, 2013, 2021). All independent variables measured on a binary 0 to 1 scale. Dining alone indicates eating all meals alone the previous day. Standard errors in parentheses. Data weighted to be nationally representative. *** p<.01, ** p<.05, * p<.1 | | | | | |
|  | | | | | |
